# Exploring 4,7-Disubstituted Pyrimido[4,5-*d*]pyrimidines as Antiviral and Anticancer Agents

**DOI:** 10.3390/molecules29235549

**Published:** 2024-11-25

**Authors:** Eleftheria A. Georgiou, Konstantinos Paraskevas, Christina Koutra, Leentje Persoons, Dominique Schols, Steven De Jonghe, Ioannis K. Kostakis

**Affiliations:** 1 Department of Pharmacy, Division of Pharmaceutical Chemistry, National and Kapodistrian University of Athens, Panepistimiopolis, Zografou, 15771 Athens, Greece; elgeorgiou@pharm.uoa.gr (E.A.G.); kon.paraskevas@outlook.com (K.P.); 2 Department of Pharmacy, Division of Pharmacognosy and Natural Products Chemistry, National and Kapodistrian University of Athens, Panepistimiopolis, Zografou, 15771 Athens, Greece; ckoutral@pharm.uoa.gr; 3 Molecular Genetics and Therapeutics in Virology and Oncology Research Group, Department of Microbiology, Immunology and Transplantation, Rega Institute for Medical Research, KU Leuven, Herestraat 49, P.O. Box 1043, 3000 Leuven, Belgium; leentje.persoons@kuleuven.be; 4 Molecular Structural and Translational Virology Research Group, Department of Microbiology, Immunology and Transplantation, Rega Institute for Medical Research, KU Leuven, Herestraat 49, P.O. Box 1043, 3000 Leuven, Belgium; dominique.schols@kuleuven.be (D.S.); steven.dejonghe@kuleuven.be (S.D.J.)

**Keywords:** pyrimido[4,5-*d*]pyrimidines, synthesis, antiviral activity, anti-proliferative activity, HCoV-229E

## Abstract

Thirteen new 4,7-disubstituted pyrimido[4,5-*d*]pyrimidines were synthesized via a straightforward methodology starting from thiourea. The anti-proliferative activity of these compounds was evaluated across a diverse panel of eight cancer cell lines, with derivatives **7d** and **7h** showing efficacy against several hematological cancer types. Furthermore, all compounds were assessed for their antiviral potency against a panel of viruses. Compounds featuring a cyclopropylamino group and an aminoindane moiety exhibited remarkable efficacy against human coronavirus 229E (HCoV-229E). These findings highlight the pyrimidino[4,5-*d*]pyrimidine scaffold as an interesting framework for the design of novel antiviral agents against HCoVs, with compounds **7a**, **7b**, and **7f** emerging as strong candidates for further investigation.

## 1. Introduction

Quinazoline derivatives are increasingly recognized for their pivotal role in the design of anticancer drugs due to their ability to selectively target cancer cells. One of the primary mechanisms by which these compounds exert their anticancer effects is through the inhibition of receptor tyrosine kinases (RTKs), enzymes that are crucial for cancer cell proliferation and survival. Mutations and dysregulations of critical RTKs like epidermal growth factor receptor (EGFR) [1], Src kinases [2], and Numb-associated kinases (NAKs) play a significant role in cell signaling pathways [3], and their inhibition has been proven to be effective in combating various cancer types [4]. The quinazoline scaffold, with its unique structural features, facilitates interactions within the ATP-binding pockets of kinase targets, enhancing selectivity and potency.

**Figure 1:**
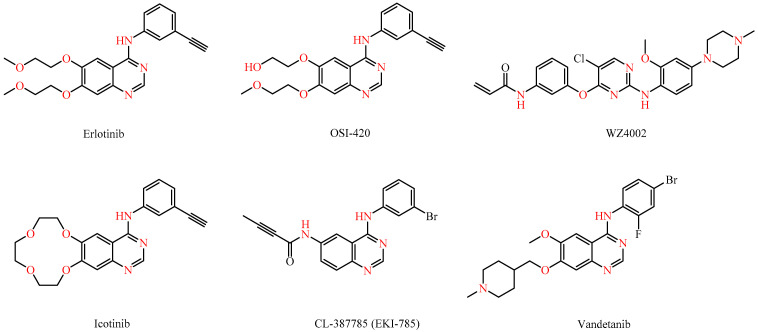
Structures of known kinase inhibitors.

Marketed drugs like gefitinib and erlotinib (Figure 1), both based on a quinazoline scaffold, have demonstrated significant efficacy in blocking EGFR in non-small cell lung cancer, offering targeted treatments with reduced toxicity. Intriguingly, EGFR is also involved in the infection processes of viruses like the influenza A virus, the hepatitis C virus (HCV), and the human cytomegalovirus (HCMV), making it a promising antiviral target [5,6,7,8,9,10,11,12,13]. As a result, erlotinib was shown to be able to inhibit the replication of many viruses by interfering with the entry and replication processes [14].

Src kinases, non-RTKs, are essential for cell motility, differentiation, and survival [15]. Targeting Src kinases is being explored for anticancer therapy and other conditions like immunotherapy and viral infections. Notably, vandetanib (Figure 1) blocks the cytokine storm in severe acute respiratory syndrome coronavirus-2 (SARS-CoV-2)-infected mice [16], while saracatinib inhibits Middle East respiratory syndrome coronavirus (MERS-CoV) [17] and other HCoVs (human coronavirus) like HCoV-229E and HCoV-OC43 at early stages of the viral cycle [18]. NAKs are implicated in a broad range of cellular functions and are implicated in various diseases, such as cancer, Parkinson’s disease, and viral infections [3]. The NAK family includes adaptor-associated kinase 1 (AAK1), cyclin G-associated kinase (GAK), BMP-2 inducible kinase (BIKE/BMP2K), and serine/threonine kinase 16 (STK16). Erlotinib, an inhibitor of GAK and AAK1, has shown broad-spectrum antiviral activity against various unrelated RNA viruses, highlighting its potential as a versatile antiviral agent [19].

As research into host kinase pathways advances, it becomes clear that understanding the broader virology context is essential, particularly in the case of HCoVs. The history of HCoVs spans from the discovery of the first strain, B814, in 1965 to the emergence of highly pathogenic strains like SARS-CoV in 2002–2003 and SARS-CoV-2 in 2019 [20]. Initially, strains like HCoV-229E and HCoV-OC43 were identified, causing mild upper respiratory tract illnesses. Over 30 strains have been identified since. In the post-SARS-CoV era, HCoV-NL63 and HCoV-HKU1 were discovered, followed by zoonotic strains like MERS-CoV and SARS-CoV-2, which lead to severe respiratory diseases [21]. While strains like HCoV-229E and HCoV-OC43 replicate mainly in the upper respiratory tract, causing mild to moderate symptoms, they can also affect the central nervous system (CNS) and may be associated with neurological diseases, though further research is needed to understand this relationship fully [22].

Since the SARS-CoV-2 pandemic, a number of small molecule drugs received marketing approval for the treatment of SARS-CoV-2 infections. These include remdesivir [23] and molnupiravir (both inhibitors of the viral polymerase) [24] as well as nirmatrelvir [25] (an inhibitor of the viral main protease). There is still a need for the discovery of novel HCoV agents. Targeting cellular host factors that are essential for viral replication offers the possibility of developing broad-spectrum antiviral agents. Since viruses rely on various host phosphorylation events, kinases activated during viral infection are potential therapeutic targets. A growing body of evidence has shown that RTKs, Src kinases, NAKs, cyclin-dependent kinases, and mitogen-activated protein kinases are of significant interest as antiviral drug targets [26,27,28,29].

The above-mentioned data highlight the essential role of kinases in both cancer and viral infections, underscoring how kinase inhibitors, such as 4-anilinoquinazolines, offer a unique therapeutic advantage. Building on this knowledge, herein we present the synthesis of 4,7-disubstituted pyrimido[4,5-*d*]pyrimidines as potential antiviral compounds. Additionally, due to their structural similarity to known EGFR inhibitors, they were also evaluated for their anticancer activity against a panel of eight cancer cell lines. These derivatives share key characteristics with 4-anilinoquinazolines, which are regarded as one of the most important classes of EGFR inhibitors. Specifically, the new derivatives feature two nitrogen atoms at positions 1 and 3, crucial for the activity since they form two essential hydrogen bonds within the ATP binding pocket of EGFR [30,31]. However, a notable difference with 4-anilinoquinazolines is the presence of two additional nitrogen atoms at positions 6 and 8. A nitrogen atom is strategically placed at position 6, where other known EGFR inhibitors typically accommodate small, electron-donating groups. Furthermore, at position 7, these new derivatives carry an amino side chain instead of the alkoxy group. The nitrogen attached to the pyrimido[4,5-*d*]pyrimidine scaffold is either secondary or tertiary, aiming to study the impact of size and substitution on the activity.

## 2. Results and Discussion

### 2.1. Chemistry

**Scheme 1:**
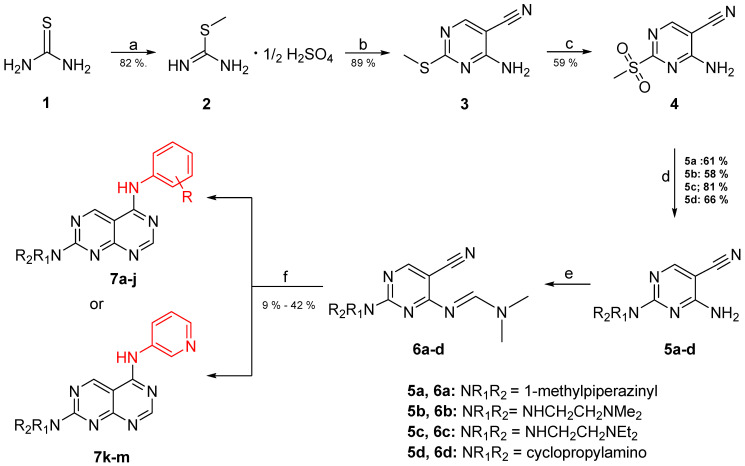
Reagents and conditions: (a) dimethyl sulfate, H_2_O, 80 °C; (b) 2-(ethoxymethylene)malononitrile, triethylamine, ethanol abs., rt; (c) *m*CPBA, CH_2_Cl_2_, 25 °C; (d) appropriate amine, THF, 25 °C; (e) DMF-DMA, toluene, rt; (f) suitable aniline, CH_3_CO_2_H, reflux.

The synthesis of the pyrimido[4,5-*d*]pyrimidines is depicted in Scheme 1, with the substituents of the final derivatives shown in Table 1. Initially, the sulfate salt of 2-methyl-2-thiopseudourea (**2**) was prepared by treatment of thiourea (**1**) with dimethyl sulfate at 80 °C (Scheme 1). Subsequently, the reaction between 2-(ethoxymethylene)malononitrile and derivative **2**, in the presence of triethylamine, afforded the desired pyrimidine **3**, which, upon oxidization by *meta*-chloroperbenzoic acid, led to the formation of the oxidized compound **4**. An aromatic nucleophilic substitution with various amines afforded the substituted pyrimidines **5a**–**5d**. Subsequent treatment with *N,N*-dimethylformamide dimethyl acetal (DMF-DMA) yielded intermediates **6a**–**d**. Finally, the desired pyrimido[4,5-*d*]pyrimidines **7a**–**7m** were obtained upon a nucleophilic addition by the appropriate aniline on the derivatives **6a**–**6d**, followed by a Dimroth rearrangement [32]. Structural elucidation of the compounds was confirmed through NMR spectroscopy, using both direct and long-range experiments. The ^1^H NMR spectra of the final compounds **7a**–**m** display two characteristic singlets in the range of 9.0–9.5 ppm and 8.3–9.0, attributed to H-2 and H-5 of the pyrimido[4,5-*d*]pyrimidine scaffold, each integrating for 1 proton. It is noticeable that these compounds exist in solution in a tautomeric equilibrium. This was evident from the ^1^H-NMR spectra, where at least 2 tautomers were observed, especially with the compounds bearing a secondary amino substituted aliphatic side chain.

### 2.2. Biological Assays

#### 2.2.1. Antiviral Activity

All new compounds were evaluated for their antiviral activity against a variety of viruses. This assessment included a DNA virus of the Herpesviridae family (herpes simplex virus-1) and several RNA viruses, namely influenza viruses (influenza A/H1N1A/Ned/378/05, influenza A/H3N2A/HK/7/87, and influenza B/Ned/537/05), yellow fever virus, Zika virus, respiratory syncytial virus (RSV), and three human coronaviruses (HCoV-229E, HCoV-OC43, and HCoV-NL63) (full data on the Supplementary Material).

The antiviral assays were conducted based on the inhibition of virus-induced cytopathic effects, and antiviral activity was quantified as the EC_50_, representing the concentration of the compound needed to reduce virus-induced cytopathogenicity by 50%. In parallel, the cytostatic activity of the compounds was investigated, defined by the CC_50_, which indicates the concentration required to diminish cell proliferation by 50% relative to untreated controls. Remdesivir, ribavirin, zanamivir, and rimantadine were included as positive controls.

As it can be derived from the data presented in Table 2, most compounds lacked antiviral activity (see also Supplementary Material). However, amino-indane (compounds **7e**–**g** and **7i**) or tetrahydronaphthalene (compounds **7a**–**b**) substitution proved crucial for antiviral activity, as these compounds demonstrated intriguing antiviral activity against HCoV-229E and HCoV-OC43, while showing no cellular toxicity. Among them, compound **7f** (amino-indane substituted) and analogs **7a** and **7b** (tetrahydronaphthalene substituted) showed the most promising results. Although the new derivatives displayed weak antiviral activity, they exhibited selective efficacy against coronaviruses 229E and OC43. Furthermore, the aliphatic chain at position 7 of the pyrimido[4,5-*d*]pyrimidine core seemed to play a critical role in antiviral effectiveness, as compounds bearing an *N*-methylpiperazinyl group (compound **7d**, **7h**, and **7k**) are inactive, in contrast to most of the compounds possessing a cyclopropylamine or longer aliphatic chains (compounds **7a**, **7b**, **7e**, and **7f**), with the exception of the tetrahydronaphthalene substituted compound **7c**, which was also inactive. The presence of an exocyclic secondary nitrogen at position 7 likely plays a key role in antiviral action. When compared to known kinase inhibitors with a 4-anilinoquinazoline structure, the new compounds performed significantly better than erlotinib and icotinib. Notably, only the irreversible EGFR inhibitors CNX-2006 and CL-387785 surpassed the antiviral activity of aminoindane substituted compound **7f**. This finding suggests that irreversible inhibition of EGFR could be a pivotal factor in the design of new, selective antiviral agents.

#### 2.2.2. Anti-Proliferative Activity

In addition, all compounds were evaluated for their anti-proliferative activities against eight diverse human cancer cell lines, namely LN-229 (glioblastoma), Capan-1 (pancreatic adenocarcinoma), HCT-116 (colorectal carcinoma), NCI-H460 (lung carcinoma), DND-41 (acute lymphoblastic leukemia), HL-60 (acute myeloid leukemia), K-562 (chronic myeloid leukemia), and Z-138 (non-Hodgkin lymphoma). Six known kinase inhibitors (erlotinib, WZ4002, OSI-420, icotinib, CNX-2006, and CL-387785) were also included in this antitumoral screening. Etoposide served as a positive control to validate the assay, while untreated cell lines were used as negative controls, establishing a baseline response in the absence of compound treatment. The results of the MTS viability assay after 72 h of treatment (expressed as 50% inhibitory concentrations (IC_50_) in µM) of the most active compounds are summarized in Table 3.

Most of the newly synthesized compounds exhibited negligible cytotoxic activity, with only five analogs demonstrating inhibitory effects on cancer cell growth, specifically against the hematological cancer cell lines. These include the tetrahydronaphthalene compounds **7a**–**b**, the aminoindane analog **7d**, and the anilino derivatives **7h–i.** The aminoindane substituted compound **7d** inhibited the growth of all tested leukemia cell lines. Likewise, the ethynylanilino analog **7h** not only inhibited leukemia cell lines but also exhibited a measurable effect on the proliferation of the glioblastoma cell line LN-229, indicating their potential for further exploration.

WZ4002 and icotinib are completely devoid of cytotoxic activity, as evidenced by CC_50_ values exceeding 100 µM. Erlotinib only showed activity against the Capan-1 (CC_50_ = 18.6 µM) and HL-60 cell lines (CC_50_ = 10.7 µM), whereas OSI-420 was only active in the Capan-1 cell line (CC_50_ = 41.7 µM). The irreversible EGFR inhibitors CNX-2006 and CL-387785 displayed potent anti-proliferative activity with CC_50_ values of less than 10 µM against the majority of the tested cell lines.

## 3. Materials and Methods

### 3.1. General Information

All commercially available reagents and solvents were obtained from Alfa Aesar (Ward Hill, MA, USA) and used directly without further purification. Melting points were determined using a Büchi apparatus (BÜCHI Labortechnik AG, Flawil, Switzerland) and are uncorrected values. One-dimensional (^1^H NMR and ^13^C NMR) and two-dimensional (COSY, NOESY, HMBC, and HSQC-DEPT135) spectra were acquired on a Bruker Avance III-600 or Bruker Avance 400 spectrometer (Karlsruhe, Germany). Chemical shifts (*δ*) are reported in ppm, while coupling constants (*J*) are given in Hz. Multiplet patterns are denoted as follows: s (singlet), d (doublet), t (triplet), q (quartet), dd (doublet of doublets), and m (multiplet). Detailed ^1^H-NMR and ^13^C-NMR data can be found in the Supplementary Material. Flash chromatography was performed using Merck silica gel (40–63 μm) with the specified solvent system, typically applying gradients of increasing polarity (Merck KGaA—Darmstadt, Germany). Reaction progress was monitored by thin-layer chromatography on pre-coated silica gel 60 F254 plates from Merck (0.25 mm thickness). Mass spectrometric analyses were conducted using a UPLC Triple TOF-MS system (UPLC: Acquity from Waters (Milford, MA 01757, USA) and SCIEX Triple TOF-MS 5600+ from Framingham, MA 01701, USA). The representative numbering for the final compounds is provided in the Supplementary Material.

### 3.2. Synthesis of Compounds ***7a**–**7m***

*Methyl carbamimidothioate sulfate* (**2**), Dimethyl sulfate (13.80 g, 110.00 mmol) was added dropwise to a mixture of thiourea (15.20 g, 200.00 mmol) in water (7 mL), and the reaction mixture was heated under reflux for 1 h. Upon completion of the reaction, the mixture was cooled to room temperature, and ethanol 95% (20 mL) was added. The solution was then cooled to 0 °C and filtered. The filter cake was washed with cold ethanol (10 mL), and the white solid was dried over phosphorus pentoxide to yield compound **2** (23 g), which was used for the next step without further purification [33]. Yield = 82%. m.p.: 233–235 °C (EtOH). ^1^H NMR (400 MHz, DMSO-*d*_6_) δ (ppm) 8.82 (brs, D_2_O exchang., 4H), 2.58 (s, 3H).

*4-Amino-2-(methylthio)pyrimidine-5-carbonitrile* (**3**), To a solution of compound **2** (10 g, 3.6 mol) in absolute ethanol (65 mL), 2-(ethoxymethylene)malononitrile (8.79 g, 7.2 mol) and triethylamine (12 mL, 8.64 mol) were added. The resulting mixture was stirred at room temperature for 2 h. Upon completion of the reaction, the precipitate was filtered and washed with ethanol to yield the title compound **3** (5.4 g). Yield = 89%. ^1^H NMR and ^13^C NMR are in agreement with the reported data [34].

*2-Amino-4-(methylsulfonyl)benzonitrile* (**4**), A suspension of 4-amino-2-(methylthio)pyrimidine-5-carbonitrile (500 mg, 3.01 mmol, **3**) and m-chloroperbenzoic acid (1.7 g, 9.91 mmol) in dry CH_2_Cl_2_ (30 mL) was stirred at room temperature for 3 h. After completion of the reaction, THF was added (20 mL), and the resulting precipitate was filtered and washed with THF to provide the title compound **4** (351 mg). Yield = 59%. m.p.: 211–214 °C (EtOAc). ^1^H NMR (600 MHz, DMSO-*d*_6_) δ (ppm) 8.88 (brs, D_2_O exchang., 1H), 8.82 (s, 1H), 8.43 (brs, D_2_O exchang., 1H), 3.31 (s, 3H). ^13^C NMR (151 MHz, DMSO-*d*_6_) δ (ppm) 166.8, 162.9, 162.4, 114.2, 91.9, 38.6.

*4-Amino-2-(4-methylpiperazin-1-yl)pyrimidine-5-carbonitrile* (**5a**), To a mixture of 4-amino-2-(methylthio)pyrimidine-5-carbonitrile (1.5 g, 7.57 mmol, **4**) in dry THF (10 mL), 1-methylpiperazine (800 mg, 7.6 mmol) was added, and the resulting mixture was stirred at room temperature for 14 h. Upon completion of the reaction, the mixture was evaporated to dryness, and the title compound **5** was purified by column chromatography (silica gel, CH_2_Cl_2_/MeOH: 50/1–20/1) to afford 532 mg of the title compound. Yield = 61%. m.p.: 197 °C (EtOH). ^1^H NMR (400 MHz, CD_3_OD) δ (ppm) 8.18 (s, 1H), 3.85 (m, 4H), 2.46 (m, 4H), 1.45 (s, 3H). ^13^C NMR (151 MHz, DMSO-*d*_6_) δ (ppm) 162.8, 161.9, 160.6, 117.2, 78.3, 54.3, 45.6, 43.0.

*4-Amino-2-((2-(dimethylamino)ethyl)amino)pyrimidine-5-carbonitrile* (**5b**), The synthetic procedure for this compound is analogous to that of **5a**, using cyclopropylamine as the starting material. Yield = 58%. ^1^H NMR (600 MHz, DMSO-*d*_6_) δ (ppm) 8.30 (minor) (s, 0.06H), 8.23 (minor) (s, 0.7H), 8.13 (major) (s, 1H), 7.31–6.95 (minor + major) (brs, D_2_O exchang., 5.28H), 3.35–3.29 (m, 2H), 2.44–2.39 (m, 2H), 2.19 (s, 6H). ^13^C NMR (151 MHz, DMSO-*d*_6_) δ (ppm) 170.1, 163.3, 162.6, 162.2, 161.8, 117.5, 117.3, 79.3, 77.6, 58.0, 57.8, 45.0, 38.3.

*4-Amino-2-((2-(diethylamino)ethyl)amino)pyrimidine-5-carbonitrile* (**5c**), The synthetic procedure for this compound is analogous to that of **5a**, using 2-(dimethylamino)propyl)amine as the starting material. Yield = 81%. m.p.: 150–151 °C (CH_2_Cl_2_/Et_2_O). ^1^H NMR (400 MHz, CDCl_3_) δ (ppm) 8.25 (minor) (s, 0.6H), 8.16 (major) (s, 1H), 6.04 (minor +major) (brs,D_2_O exchang., 1.6H), 3.42 (minor + major) (m, 3.2H), 2.62 (minor + major) (t, *J* = 5.8 Hz, 3.2H), 2.55 (minor + major) (q, *J* = 7.1 Hz, 6.4H), 1.65 (minor +major) (brs,D_2_O exchang., 3.2H), 1.02 (minor + major) (t, *J* = 7.1 Hz, 9.6H). ^13^C NMR (151 MHz, DMSO-*d*_6_/D_2_O—9/1, 7 °C) δ (ppm) 163.4, 162.8, 162.6, 162.4, 162.3, 162.2, 117.7, 117.5, 80.1, 78.3, 50.3, 49.9, 49.7, 46.8, 46.8, 36.4, 36.31, 9.49.

*4-Amino-2-(cyclopropylamino)pyrimidine-5-carbonitrile* (**5d**), The synthetic procedure for this compound is analogous to that of **5a**, using cyclopropylamine starting material. Yield = 66%. m.p.: 114 °C (CH_2_Cl_2_/Et_2_O). ^1^H NMR (600 MHz, DMSO-*d*_6_/D_2_O—9/1, 7 °C) δ (ppm) 8.25 (minor) (s, 0.7H), 8.15 (major), (s, 1H), 3.27 (minor) (t, *J* = 6.8 Hz, 1.4H), 3.24 (major) (t, *J* = 6.8 Hz, 2H), 2.60 (minor + major) (brs, 6.8H), 2.56 (minor + major) (brs,D_2_Oexchang., 3.4H), 1.65 (minor +major) (m, 3.4H), 0.99 (minor + major) (t, *J* = 7.1 Hz, 10.2H). ^13^C NMR (151 MHz, DMSO-*d*_6_/D_2_O—9/1, 7 °C) δ (ppm) 163.4, 162.7, 162.6, 162.4, 162.2, 118.0, 117.8, 79.3, 77.6, 50.0, 49.8, 46.4, 25.9, 25.5, 11.1.

*N^2^-[2-(Dimethylamino)ethyl]-N^5^-(5,6,7,8-tetrahydronaphthalen-1-yl)pyrimido[4,5-d]pyrimidine-2,5-diamine* (**7a**), To a suspension of compound **5a** (71 mg, 0.32 mmol) in dry toluene (15 mL), N, N-dimethylformamide-dimethylacetal (42.4 μL, 0.32 mmol) was added, and the resulting mixture was stirred at 110 °C for 4 h. After completion of the reaction, the resulting mixture was vacuum-evaporated. The oily residue was purified by column chromatography (silica gel, CH_2_ Cl_2_/MeOH: 50/1–20/1) to afford **6a** (70 mg) as a mixture of E/Z isomers that was added without any further purification to a solution of amine 5,6,7,8-tetrahydronaphthalen-1-amine (39.50 mg, 0.27 mmol) in glacial acetic acid (2 mL). The resulting mixture was stirred under reflux for 2.5 h, and after completion of the reaction, it was then evaporated to dryness. The oily residue was purified by column chromatography (silica gel, CH_2_Cl_2_/MeOH: 20/1.15/1–12/1 to afford **7a** (12.04 mg). Yield = 15%. m.p.: 176.3 °C (MeOH/CH_2_Cl_2_^1^H NMR (400 MHz, CDCl_3_/MeOD) δ 8.80 (s, 1H), 8.63 (s, 1H), 7.37–7.31 (m, 1H), 7.18 (t, *J* = 7.7 Hz, 1H), 7.07 (dd, *J* = 7.5, 1.4 Hz, 1H), 6.28 (s, 1H), 3.64 (d, *J* = 5.4 Hz, 2H), 2.87–2.78 (m, 2H), 2.66 (d, *J* = 5.8 Hz, 2H), 2.57 (t, *J* = 6.0 Hz, 2H), 2.27 (s, 7H), 1.67 (s, 6H). ^13^C NMR (151 MHz, CDCl_3_/MeOD) δ (ppm) 164.3, 162.7, 160.6, 158.1, 139.1, 135.7, 133.8, 128./, 126.1, 124.2, 100.7, 57.9, 45.1, 38.7, 29.7, 25.1, 22.8, 22.7. HRMS (ESI^+^) *m*/*z* 364.2238 (calcd for C_20_H_26_N_7_^+^, 364.2244).

*N^2^-[2-(Diethylamino)ethyl]-N^5^-(5,6,7,8-tetrahydronaphthalen-1-yl)pyrimido[4,5-d]pyrimidine-2,5-diamine* (**7b**), The synthetic procedure for this compound is analogous to that of **7a**. Yield = 26%. m.p.: 181.3 °C (CH_2_Cl_2_). ^1^H NMR (600 MHz, CDCl_3_/MeOD) δ (ppm) 9.27 (s, 1H), 8.33 (s, 1H), 7.16 (t, *J* = 7.7 Hz, 1H), 7.08 (dd, *J* = 7.7, 2.9 Hz, 2H), 4.40 (s, 2H), 3.66 (t, *J* = 6.7 Hz, 2H), 2.89 (s, 2H), 2.83 (t, *J* = 6.0 Hz, 2H), 2.65 (t, *J* = 6.0 Hz, 3H), 1.78 (q, *J* = 6.7 Hz, 4H), 1.14 (t, *J* = 7.4 Hz, 6H). ^13^C NMR (151 MHz, CDCl_3_/MeOD) δ (ppm) 163.7, 161.9, 160.3, 158.7, 154.0, 147.4, 138.7, 135.5, 134.4, 128.5, 125.7, 124.6, 97.3, 51.4, 47.0, 38.1, 38.1, 29.4, 29.4, 24.8, 22.6, 10.1. HRMS (ESI^+^) *m*/*z* 392.2565 (calcd for C_22_H_30_N_7_^+^, 392.2557).

*N^2^-cyclopropyl-N^5^-(5,6,7,8-tetrahydronaphthalen-1-yl)pyrimido[4,5-d]pyrimidine-2,5-diamine* (**7c**), The synthetic procedure for this compound is analogous to that of **7a**. Yield = 40%. m.p.: 189 °C (EtOAc). ^1^H NMR (600 MHz, DMSO-*d*_6_) δ (ppm) ^1^H NMR (600 MHz,) δ (ppm) 9.75 (minor) (s, 0.3H), 9.65 (major) (s, 1H), 9.47 (minor) (s, 0.2H), 9.35 (major) (s, 1H), 8.26 (s, 1H), 8.01 (d, J = 4.4 Hz, 1H), 7.07 (t, *J* = 7.5 Hz, 1H), 7.02 (d, *J* = 7.6 Hz, 1H), 6.97 (d, *J* = 6.8 Hz, 1H), 2.82–2.74 (m, 2H), 2.70 (t, *J* = 6.3 Hz, 5H), 2.42 (p, *J* = 1.9 Hz, 4H), 1.62 (q, *J* = 7.0 Hz, 11H), 0.81–0.57 (m, 4H), 0.47 (d, *J* = 5.3 Hz, 5H). ^13^C NMR (151 MHz, DMSO-*d*_6_) δ (ppm) 164.4, 163.9, 162.0, 159.7, 158.4, 137.8, 136.2, 134.0, 127.5, 125.4, 125.1, 100.1, 29.1, 24.5, 24.0, 22.4, 22.3, 6.4, 6.2.HRMS (ESI^+^) *m*/*z* 333.1828 (calcd for C_19_H_21_N_6_^+^, 333.1822).

*N-(2,3-dihydro-1H-inden-4-yl)-7-(4-methylpiperazin-1-yl)pyrimido[4,5-d]pyrimidin-4-amine* (**7d**), The synthetic procedure for this compound is analogous to that of **7a**. Yield = 43%. m.p.: >250 °C (EtOAc). ^1^H NMR (600 MHz, CDCl_3_) δ (ppm) 9.02 (s, 1H), 8.63 (s, 1H), 7.46 (d, *J* = 7.8 Hz, 1H), 7.22 (t, *J* = 7.6 Hz, 1H), 7.16 (dd, *J* = 7.4, 1.0 Hz, 1H), 4.17–4.09 (m, 4H), 2.99 (t, *J* = 7.4 Hz, 2H), 2.85 (t, *J* = 7.4 Hz, 2H), 2.65 (s, 4H), 2.44 (s, 3H), 2.10 (p, *J* = 7.5 Hz, 2H), 2.04 (d, *J* = 2.5 Hz, 1H). ^13^C NMR (151 MHz, CDCl_3_) δ (ppm) 164.3, 162.77, 162.1, 159.2, 157.4, 146.5, 138.4, 133.8, 127.7, 122.9, 122.1, 100.5, 54.5, 45.5, 43.2, 33.4, 31.0, 25.1. HRMS (ESI^+^) *m*/*z* 362.2096 (calcd for C_20_H_24_N_7_^+^, 362.2088).

*N^5^-(2,3-dihydro-1H-inden-4-yl)-N^2^-(2-(dimethylamino)ethyl)pyrimido[4,5-d]pyrimidine-2,5-diamine* (**7e**), The synthetic procedure for this compound is analogous to that of **7a**. Yield = 12%. m.p.: 180.9 °C. (EtOAc). ^1^H NMR (400 MHz, CDCl_3_) δ (ppm) 8.99 (s, 1H), 8.65 (s, 1H), 7.49 (d, *J* = 7.8 Hz, 1H), 7.21 (t, *J* = 7.6 Hz, 1H 7.15 (d, 1H), 6.40 (s, 1H), 3.63 (q, *J* = 6.5, 6.1 Hz, 2H), 2.99 (t, *J* = 7.5 Hz, 2H), 2.85 (t, *J* = 7.4 Hz, 2H), 2.57 (t, *J* = 5.9 Hz, 2H), 2.28 (s, 6H), 2.10 (p, *J* = 7.5 Hz, 2H). ^13^C NMR (151 MHz, CDCl_3_) δ (ppm) 164.3, 162.1, 162.8, 161.6, 159.4, 158.1, 146.2, 133.6, 127.4, 123.0, 122.7, 100.9, 57.8, 45.0, 33.2, 31.2, 29.8, 25.1. HRMS (ESI^+^) *m*/*z* 350.2081 (calcd for C_19_H_24_N_7_^+^, 350.2088).

*N^5^-(2,3-dihydro-1H-inden-4-yl)-N^2^-[2-(diethylamino)ethyl]pyrimido[4,5-d]pyrimidine-2,5-diamine* (**7f**), The synthetic procedure for this compound is analogous to that of **7a**. Yield = 10%. m.p.: 172.9 °C (EtOAc/diethyl ether). ^1^H NMR (600 MHz, CDCl_3_) δ (ppm) 9.11 (s, 1H), 8.55 (d, *J* = 4.6 Hz, 1H), 7.28 (d, *J* = 7.8 Hz, 1H), 7.18 (t, *J* = 7.6 Hz, 1H), 7.14 (d, *J* = 7.4 Hz, 1H), 3.68 (t, *J* = 5.8 Hz, 2H), 2.96 (t, *J* = 7.5 Hz, 2H), 2.80 (t, *J* = 7.4 Hz, 2H), 2.73 (d, *J* = 5.9 Hz, 2H), 2.38 (s, 6H). ^13^C NMR (151 MHz, CDCl_3_) δ (ppm) 168.9, 163.6, 163.6, 159.9, 146.2, 140.0, 133.6, 127.2, 123.4, 123.0, 106.9, 47.2, 37.5, 33.5, 31.6, 25.2, 9.0. HRMS (ESI^+^) *m*/*z* 378.2391 (calcd for C_21_H_28_N_7_^+^, 378.2401).

*N^2^-cyclopropyl-N^5^-(2,3-dihydro-1H-inden-4-yl)pyrimido[4,5-d]pyrimidine-2,5-diamine* (**7g**), The synthetic procedure for this compound is analogous to that of **7a**. Yield = 42%. m.p.: 290 °C (EtOAc). ^1^H NMR (600 MHz, DMSO-*d*_6_)/CDCl_3_) δ (ppm) 10.50 (s, 1H), 9.49 (s, 1H), 8.49 (s, 1H), 7.45 (s, 1H), 7.20 (d, *J* = 6.7 Hz, 1H), 7.16 (dd, *J* = 6.8, 2.3 Hz, 1H), 2.94 (t, *J* = 7.4 Hz, 1H), 2.89 (s, 1H), 2.77 (t, *J* = 7.4 Hz, 1H), 1.99 (p, *J* = 7.5 Hz, 1H), 0.77 (dd, *J* = 7.0, 2.3 Hz, 1H), 0.59 (s, 1H). ^13^C NMR (151 MHz DMSO-*d*_6_)/CDCl_3_) δ (ppm) 163.3, 162.4, 161.5, 159.7, 144.8, 133.9, 126.7, 120.2, 118.6, 117.3, 36.0, 32.7, 30.1, 24.4, 23.8, 6.2. HRMS (ESI^+^) *m*/*z* 319.1674 (calcd for C_18_H_19_N_6_^+^, 319.1666).

*N-(3-ethynylphenyl)-7-(4-methylpiperazin-1-yl)pyrimido[4,5-d]pyrimidin-4-amine* (**7h**), The synthetic procedure for this compound is analogous to that of **7a**. Yield = 9%. m.p.: 290 °C (MeOH). ^1^H NMR (400 MHz,) δ (ppm) 9.98 (s, 1H), 9.60 (s, 1H), 8.53 (s, 1H), 7.63 (dd, *J* = 8.1, 1.9 Hz, 1H), 7.58–7.47 (m, 1H), 7.38–7.17 (m, 1H), 7.00 (dd, *J* = 7.8, 1.7 Hz, 1H), 3.90 (s, 5H), 2.39 (t, *J* = 5.1 Hz, 5H), 2.23 (s, 3H). ^13^C NMR (151 MHz, DMSO-*d*_6_) δ (ppm) 163.5, 162.1, 161.8, 158.9, 158.5, 144.3, 138.5, 128.4, 123.7, 121.8, 120.1, 100.1, 83.1, 80.6, 54.4, 45.7, 43.4 HRMS (ESI^+^) *m*/*z* 346.1782 (calcd for C_19_H_20_N_7_^+^, 346.1775).

*N^2^-[2-(diethylamino)ethyl]-N^5^-(3-ethynylphenyl)pyrimido[4,5-d]pyrimidine-2,5-diamine* (**7i**), The synthetic procedure for this compound is analogous to that of **7a**. Yield = 57%. m.p.: 182 °C (EtOAc). ^1^H NMR (600 MHz, DMSO-*d*_6_/D_2_O—9/1, 7 °C) δ (ppm) 9.61 (minor) (s, 0.3H), 9.51 (major) (s, 1H), 8.58 (major) (s, 1H), 8.56 (minor) (s, 0.3H), 8.00 (minor) (s, 03H), 7.99 (major) (s, 1H), 7.81 (minor + major) (d, *J* = 7.9 Hz, 1.3H), 7.41 (minor + major) (t, *J* = 7.9 Hz, 1.3H), 7.24 (minor + major) (d, *J* = 7.6 Hz, 1.3H), 4.25 (minor + major) (s, 1.3H), 3.47 (minor) (t, *J* = 7.1 Hz, 0.6H), 3.42 (major) (t, *J* = 7.1 Hz, 2H), 2.61 (major) (t, *J* = 7.1 Hz, 2H), 2.57 (minor) (t, *J* = 7.1 Hz, 0.6H), 2.52 (minor + major) (m, 5.2H), 0.97 (minor + major) (t, *J* = 7.1 Hz, 7.8H). ^13^C NMR (151 MHz, DMSO-*d*_6_/D_2_O—9/1, 7 °C) δ (ppm) 163.9, 163.4, 161.9, 159.1, 158.3, 139.2, 129.3, 127.2, 125.0, 122.8, 122.0, 100.4, 83.6, 81.0, 51.0, 46.6, 39.9, 31.0, 12.0. HRMS (ESI^+^) *m*/*z* 362.2096 (calcd for C_20_H_24_N_7_^+^, 362.2088).

*N^2^-cyclopropyl-N^5^-(3-ethynylphenyl)pyrimido[4,5-d]pyrimidine-2,5*-diamine (**7j**), The synthetic procedure for this compound is analogous to that of **7a**. Yield = 11%. m.p.: 296 °C (EtOAc). ^1^H NMR (400 MHz, DMSO-*d*_6_) δ (ppm) 9.97 (minor) (s, 0.2H), 9.91 (major) (s, 1H), 9.63 (minor) (s, 0.2H), 9.53 (major) (s, 1H), 8.54 (minor + major) (s, D_2_O exchang., 0.6H), 8.17 (minor + major) (d, *J* = 3.4 Hz, 1.2H), 7.65 (minor + major) (d, *J* = 8.0 Hz, 1.2H), 7.57 (minor + major) (s, 1.2H), 7.30 (minor + major) (t, *J* = 7.8 Hz, 1.2H), 7.00 (minor + major) (d, *J* = 7.5 Hz, 1.2H), 4.25 (minor + major) (s, 1.2H), 2.90 (minor + major) (m, 1.2H), 0.75 (minor + major) (m, 2.4H), 0.57 (minor + major) (brs, 2.4H). ^13^C NMR (151 MHz, DMSO-*d*_6_) δ (ppm) 164.4, 163.9, 161.7, 159.5, 158.5, 158.3, 144.1, 139.1, 138.7, 129.1, 128.5, 127.0, 125.1, 124.9, 123.5, 122.9, 122.6, 121.9, 121.6, 119.9, 100.9, 100.6, 83.4, 80.8, 24.1, 6.2. HRMS (ESI^+^) *m*/*z* 303.1358 (calcd for C_17_H_15_N_6_^+^, 303.1353).

*7-(4-methylpiperazin-1-yl)-N-(pyridin-3-yl)pyrimido[4,5-d]pyrimidin-4-amine* (**7k**), The synthetic procedure for this compound is analogous to that of **7a**. Yield = 20%. m.p.: >300 °C (MeOH).). ^1^H NMR (600 MHz, DMSO-*d*_6_) δ (ppm) 10.17 (s, 1H), 9.61 (s, 1H), 8.93 (s, 1H), 8.57 (s, 1H), 8.35 (d, *J* = 2.7 Hz, 1H), 8.23 (d, *J* = 6.8 Hz, 1H), 7.44 (dd, *J* = 6.8 Hz, 2.7 Hz, 1H), 3.92 (brs, 4H), 2.41 (m, 4H), 2.24 (s, 3H). ^13^C NMR (151 MHz, DMSO-*d*_6_) δ (ppm) 163.5, 161.9, 161.8, 159.0, 158.5, 144.7, 143.7, 135.4, 129.4, 123.4, 100.2, 54.4, 45.7, 43.5. HRMS (ESI^+^) *m*/*z* 323.1721 (calcd for C_16_H_19_N_8_^+^, 323.1727).

*N^2^-[2-(diethylamino)ethyl]-N^5^-(pyridin-3-yl)pyrimido[4,5-d]pyrimidine-2,5*-diamine (**7l**), The synthetic procedure for this compound is analogous to that of **7a**. Yield = 38%. m.p.: 220–224 °C (EtOAc). ^1^H NMR (600 MHz, DMSO-*d*_6_/D_2_O—9/1, 7 °C) δ (ppm) 10.11 (minor + major) (s, 1.3H), 9.63 (minor) (s, 0.3H), 9.54 (major) (s, 1H), 8.93 (minor + major) (s, 1.3H), 8.56 (minor + major) (s, 1.3H), 8.34 (minor + major) (s, 1.3H), 8.22 (minor + major) (d, *J* = 7.4 Hz, 1.3H), 7.85 (major) (t, *J* = 5.5, 1H), 7.82 (minor) (brs, *J* = 0.3H), 7.43 (minor + major) (dt, *J* = 13.8 Hz, 6.9 Hz, 1.3H), 3.49 (minor) (m, 0.6H), 3.45 (major) (m, 2H), 2.65 (major) (t, *J* = 6.8 Hz, 2H), 2.61 (minor) (m, 0.6H), 2.55 (minor + major) (m, 5.2H), 0.99 (minor + major) (t, *J* = 7.1 Hz, 7.8H). ^13^C NMR (151 MHz, DMSO-*d*_6_/D_2_O—9/1, 7 °C) δ (ppm) 164.1, 163.5, 162.4, 162.1, 159.5, 158.7, 151.0, 150.2, 145.1, 143.8, 137.5, 135.9, 131.3, 130.3, 129.2, 128.3, 128.1, 124.2, 120.3, 118.7, 100.1, 92.6, 51.5, 50.9, 50.7, 38.4, 37.9, 19.9, 16.0, 11.6, 11.5, 11.3. HRMS (ESI^+^) *m*/*z* 339.2045 (calcd for C_17_H_23_N_8_^+^, 339.2040).

*N^2^-cyclopropyl-N^5^-(pyridin-3-yl)pyrimido[4,5-d]pyrimidine-2,5-diamine* (**7m**), The synthetic procedure for this compound is analogous to that of **7a**. Yield = 42%. m.p.: 243 °C (MeOH). ^1^H NMR (400 MHz, DMSO-*d*_6_) δ (ppm) 10.10 (major) (s, 1H), 9.64 (minor) (s, 0.2H), 9.53 (major) (s, 1H), 8.92 (minor + major) (s, 1.2H), 8.58 (minor + major) (s, 1.2H), 8.33 (minor + major) (d, *J* = 4.7 Hz, 1.2H), 8.23 (minor + major) (d, *J* = 3.8 Hz, 1.2H), 8.23 (minor + major) (s, 1.2H), 7.42 (minor + major) (dd, *J* = 4.7 Hz, 3.8 Hz, 1.2H), 2.89 (s, 1H), 0.56 (m, 2H), 0.48 (m, 2H). ^13^C NMR (151 MHz, DMSO-*d*_6_) δ (ppm) 164.4, 163.8, 163.3, 162.4, 161.6, 158.5, 158.4, 144.6, 143.5, 135.6, 129.2, 123.4, 117.5, 100.5, 24.0, 23.8, 6.2. HRMS (ESI^+^) *m*/*z* 280.1312 (calcd for C_14_H_14_N_7_^+^, 280.1305).

### 3.3. Biological Assays and Experiments

For all biological assays, the test compounds and reference drugs were dissolved in DMSO at a concentration of 25 mM.

*Anti-proliferative Activity*

Cancer Cell Lines

The human cancer cell lines Capan-1, HCT-116, NCI-H460, LN-229, HL-60, K-562, and Z-138 were obtained from the American Type Culture Collection (ATCC, Manassas, VA, USA). The DND-41 cell line was sourced from the Deutsche Sammlung von Mikroorganismen und Zellkulturen (DSMZ Leibniz-Institut, Braunschweig, Germany). All cell lines were cultured according to the suppliers’ recommendations. Culture media were purchased from Gibco (Gibco Life Technologies, Merelbeke, Belgium) and supplemented with 10% fetal bovine serum (HyClone, Cytiva, MA, USA).

Proliferation Assays

Adherent cell lines were seeded at densities ranging from 500 to 1500 cells per well in 384-well plates (Greiner Bio-One, Vilvoorde, Belgium). Following overnight incubation, cells were treated with seven different concentrations of the test compounds, ranging from 100 to 0.006 µM. Etoposide was included as a positive control to validate the assay conditions. Untreated cell lines (i.e., without compound treatment) were used as negative controls.

Suspension cell lines were seeded at densities ranging from 2500 to 5000 cells per well in 384-well culture plates containing the test compounds at the same concentration points. All cell lines were incubated for 72 h with compounds and then analyzed using the CellTiter 96^®^ AQueous One Solution Cell Proliferation Assay (MTS) reagent (Promega, Leiden, The Netherlands) according to the manufacturer’s instructions. Absorbance of the samples was measured at 490 nm using a SpectraMax Plus 384 (Molecular Devices, CA, USA), and OD values were used to calculate the 50% inhibitory concentration (IC_50_). Compounds were tested in two independent experiments [35].

*Antiviral Activity*

Host Cell Lines

HEL 299 (ATCC CCL-137; human lung fibroblast), Hep3B (ATCC HB-8064; human hepatocellular carcinoma), HEp-2 (ATCC CCL-23; human cervical adenocarcinoma), VeroE6 (ATCC CRL-1586 ; African green monkey kidney cells), and MDCK (Madin-Darby canine kidney cells; a kind gift from M. Matrosovich, Marburg, Germany) were maintained in Dulbecco’s Modified Eagle Medium (DMEM, Gibco Life Technologies, Merelbeke, Belgium) supplemented with 8% heat-inactivated fetal bovine serum (HyClone, GE Healthcare Life Sciences, Diegem, Belgium), 0.075% sodium bicarbonate (Gibco Life Technologies), and 1mM sodium pyruvate (Gibco Life Technologies) and maintained at 37 °C under 5% CO_2_.

Antiviral CPE Reduction Assays

Antiviral assays toward herpes simplex virus-1 (HSV-1 KOS) and human coronavirus (HCoV-229E and -OC43) in HEL cell cultures, respiratory syncytial virus A in HEp-2 cells, yellow fever virus, Zika virus in VeroE6 cell cultures, human coronavirus (HCoV-NL63) in Hep3B cell cultures and influenza A/H1N1 (A/Ned/378/05), influenza A/H3N2 (A/HK/7/87), and influenza B (B/Ned/537/05) in MDCK cell cultures were performed. On the day of the infection, the growth medium was aspirated and replaced by serial dilutions of the test compounds in UltraMDCK serum-free medium for influenza virus or DMEM with 4% FBS for all other viruses. The virus was then added and diluted to obtain a viral input of 100 CCID50 (CCID50 being the virus dose able to infect 50% of the cell cultures). Mock-treated cultures receiving solely the test compounds were included to determine the cytotoxicity. After 3 to 7 days of incubation, the virus-induced cytopathogenic effect was measured colorimetrically by the formazan-based 3-(4,5-dimethylthiazol-2-yl)-5-(3-carboxymethoxyphenyl)-2-(4-sulfophenyl)-2H-tetrazolium (MTS) cell viability assay (CellTiter 96 AQueous One Solution Cell Proliferation Assay from Promega Leiden, The Netherlands),, and the antiviral activity was expressed as the 50% effective concentration (EC50). In parallel, the 50% cytotoxic concentration (CC_50_) was derived from the mock-infected cells. Data were obtained in two or three independent experiments. The activities were compared with the activities of reference antiviral drugs: remdesivir, ribavirin, zanamivir, and rimantadine.

## 4. Conclusions

In conclusion, most of the newly synthesized compounds demonstrated limited antiviral activity, with certain derivatives showing promise against human coronaviruses HCoV-229E and HCoV-OC43. Compounds **7a**, **7b**, and **7f** exhibited selective antiviral effects, with the amino-indane and tetrahydronaphthalene moieties appearing essential for activity. The role of the aliphatic chain and the presence of secondary nitrogen were also critical for antiviral efficacy. Despite the low overall activity, these derivatives outperformed known EGFR inhibitors like erlotinib and icotinib, highlighting their potential in targeting viral infections. Additionally, compounds **7a**, **7d**, and **7i** showed moderate antiproliferative effects on selected cancer cell lines. This indicates the potential of the pyrimido[4,5-*d*]pyrimidine scaffold for further development in both antiviral and anti-proliferative contexts. The structural features identified in this study offer valuable insights for the future design of more potent antiviral and antitumoral agents.

## Figures and Tables

**Table 1:** Substituents at position 4 and 7 of pyrimido[4,5-*d*]pyrimidines **7a**–**7m**.

Compd.	Core	NR_1_R_2_	Compd.	Core	NR_1_R_2_
**7a**	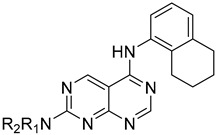	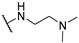	**7h**	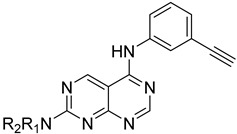	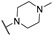
**7b**		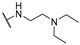	**7i**		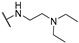
**7c**		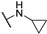	**7j**		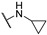
**7d**	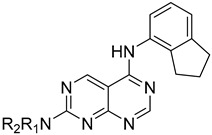	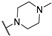	**7k**	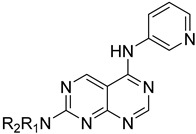	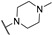
**7e**		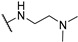	**7l**		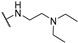
**7f**		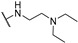	**7m**		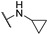
**7g**		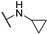			

**Table 2:** Broad-spectrum antiviral and cytotoxicity evaluation of pyrimido[4,5-d]pyrimidines **7a**–**m** and six EGFR inhibitors. Results (in µM) are presented as the mean ± standard deviation of two or three independent experiments.

	Antiviral Activity (EC_50_)							Cytotoxicity (CC_50_)				
Compd.	HCoV			Influenza			RSV	HEL299	Hep3B	MDCK	Hep2	VeroE6
	229E	OC43	NL63	H1N1	H3N2	B	A Long					
	HEL299		Hep3B	MDCK			Hep2					
**7a**	15.8 ± 0.9	27.7 ± 0.6	>50	>50	>50	>50	>50	>50	>50	>50	>50	>50
**7b**	11.0 ± 2.7	34.7 ± 16.7	>50	>50	>50	>50	>50	>50	>50	>50	>50	>50
**7c**	>50	>50	>50	>50	>50	>50	>50	>50	>50	>50	>50	>50
**7d**	>50	>50	>50	>50	>50	>50	>50	>50	>50	>50	>50	>50
**7e**	20.6 ± 0.3	>50	>50	>50	>50	>50	>50	>50	>50	>50	>50	>50
**7f**	3.9 ± 2.9	37.3 ± 15.1	>50	>50	>50	>50	>50	>50	>50	>50	>50	>50
**7g**	≥30.5	>50	>50	>50	>50	>50	>50	>50	>50	>50	>50	>50
**7h**	>50	>50	>50	>50	>50	>50	>50	>50	>50	>50	29.6 ± 1.6	>50
**7i**	19.3 ±6.6	≥39.8	>50	>50	>50	>50	>50	>50	25.7 ± 0.6	>50	>50	>50
**7j**	>50	>50	>50	>50	>50	>50	>50	>50	>50	>50	>50	>50
**7k**	>50	>50	>50	>50	>50	>50	>50	>50	>50	>50	>50	>50
**7l**	>50	>50	>50	>50	>50	>50	>50	>50	>50	>50	>50	>50
**7m**	>50	>50	>50	>50	>50	>50	>50	>50	>50	>50	>50	>50
**Erlotinib**	22.5 ± 10	>100	>100	>100	>100	>100	>100	>100	>100	>100	>100	>100
**WZ4002**	41.8 ± 9.8	>100	>100	>100	>100	>100	>100	>100	>100	>100	>100	>100
**OSI-420**	>100	>100	>100	>100	>100	>100	>100	>100	>100	>100	41.0 ± 13.4	>100
**Icotinib**	>100	>100	>100	>100	>100	>100	>100	>100	>100	>100	>100	>100
**CNX-2006**	1.8 ± 0.2	2.2 ± 0.8	>100	>100	>100	>100	>100	11.1 ± 1.0	4.3 ± 1.8	1.7 ± 0.4	11.7 ± 0.8	9.2 ± 1.1
**CL-387785**	0.4 ± 0.2	1.8 ± 0.4	>100	>100	>100	4.5 ± 1.2	>100	>100	10.9 ± 0.6	7.1 ± 0.4	10.4 ± 1.9	>100
**Remdesivir**	0.05 ± 0.04	0.14 ± 0.007	0.14 ± 0.04	-	-	-	0.008 ± 0.001	>10	8.1 ± 2.5	-	9.0 ± 1.3	-
**Ribavirin**	38.9 ± 1.7	100.1 ± 19.0	39.9 ± 7.1	9.6 ± 1.0	7.2 ± 1.6	7.4 ± 0.8	35.9 ± 1.3	>250	165.1 ± 20.2	>250	88.8 ± 23.5	>250
**Zanamivir**	-	-	-	0.6 ± 0.3	3.0 ± 0.2	0.4 ± 0.01	-	-	-	>100	-	-
**Rimantadine**	-	-	-	1.7 ± 0.5	0.6 ± 0.07	>100	-	-	-	>100	-	-

**Table 3:** Anti-proliferative data in a panel of human cancer cell lines. Results (in µM) are presented as the mean ± standard deviation of two independent experiments.

	Anti-Proliferative Activity (IC_50_)							
Compd.	LN-229	Capan-1	HCT-116	NCI-H460	DND-41	HL-60	K-562	Z-138
**7a**	>100	>100	>100	>100	57.9 ± 2.0	>100	87.0 ± 1.3	58.9 ± 1.8
**7b**	>100	>100	>100	>100	67.1 ± 4.7	>100	>100	62.6 ± 0.8
**7c**	>100	>100	>100	>100	>100	>100	>100	>100
**7d**	>100	>100	>100	>100	80.6 ± 10.2	≥71.0	61.3 ± 2.4	56.5 ± 1.2
**7e**	>100	>100	>100	>100	>100	>100	>100	>100
**7f**	>100	>100	>100	>100	>100	>100	>100	>100
**7g**	>100	>100	>100	>100	>100	>100	>100	>100
**7h**	97.1 ± 1.5	>100	>100	>100	66.5 ± 0.8	79.1 ± 4.6	52.3 ± 4.4	52.6 ± 1.4
**7i**	>100	>100	>100	>100	62.6 ± 0.1	>100	51.8 ± 3.5	62.1 ± 0.8
**7j**	>100	>100	>100	>100	>100	>100	>100	>100
**7k**	>100	>100	>100	>100	>100	>100	>100	>100
**7l**	>100	>100	>100	>100	>100	>100	>100	>100
**7m**	>100	>100	>100	>100	>100	>100	>100	>100
**Erlotinib**	>100	18.6 ± 1.1	>100	>100	>100	10.7 ± 0.1	>100	>100
**WZ4002**	>100	>100	>100	>100	>100	>100	>100	>100
**OSI-420**	>100	41.7 ± 3.6	>100	>100	>100	>100	>100	>100
**Icotinib**	>100	>100	>100	>100	>100	>100	>100	>100
**CNX-2006**	8.2 ± 1.2	1.7 ± 0.01	6.6 ± 0.7	8.7 ± 1.9	4.5 ± 0.2	3.8 ± 1.4	11.5 ± 0.06	2.6 ± 0.5
**CL-387785**	43.8 ± 6.7	0.1 ± 0.01	1.3 ± 1.1	2.8 ± 0.6	0.9 ± 0.2	1.9 ± 0.04	65.0 ± 0.2	0.2 ± 0.1
**Etoposide**	3.0 ± 0.2	0.1 ± 0.03	1.4 ± 0.09	2.2 ± 0.8	0.1 ± 0.02	0.2 ± 0.01	6.0 ± 2.0	0.08 ± 0.03

## Data Availability

Data are contained within the article and Supplementary Materials.

## Supplementary Materials

The following are available online at https://www.mdpi.com/article/10.3390/molecules29235549/s1, antiviral and anti-proliferative activity, **^1^**H-NMR, and ^13^C-NMR are available online.
