# Supplementary material for: Exploring 4,7-Disubstituted Pyrimido[4,5-d]pyrimidines as Antiviral and Anticancer Agents
Source: Molecules. 2024 Nov 25;29(23):5549. doi: 10.3390/molecules29235549 (PMC11643962; doi:10.3390/molecules29235549)
Supplement: Supplementary file 1 [file molecules-29-05549-s001.zip › molecules-3289423-supplementary.pdf]

*Article*

# Exploring 4,7-Disubstituted Pyrimido[4,5-*d*]pyrimidines as Antiviral and Anticancer Agents

**Eleftheria A. Georgiou [1], Konstantinos Paraskevas [1], Christina Koutra [1], Leentje Persoons [2], Dominique Schols [3], Steven De Jonghe [3], and Ioannis K. Kostakis [1,*]**

[1] Department of Pharmacy, Division of Pharmaceutical Chemistry, National and Kapodistrian University of Athens, Panepistimiopolis, Zografou, 15771 Athens, Greece; elgeorgiou@pharm.uoa.gr (E.G.); kon.paraskevas@outlook.com (K.P.).

[2] Department of Microbiology, Immunology and Transplantation, Rega Institute for Medical Research,
Molecular Genetics and Therapeutics in Virology and Oncology Research Group, KU Leuven, Herestraat 49, Box 1043, 3000 Leuven, Belgium; leentje.persoons@kuleuven.be (L.P.).

[3] Department of Microbiology, Immunology and Transplantation, Rega Institute for Medical Research, Molecular Structural and Translational Virology Research Group, KU Leuven, Herestraat 49, Box 1043, 3000 Leuven, Belgium. dominique.schols@kuleuven.be (D.S.); steven.dejonghe@kuleuven.be (S.D.J.)

* Correspondence: ikkostakis@pharm.uoa.gr; Tel.: +30-210-727-4212

## Contents

**Figure S1:** $^{1}$H NMR spectrum of compound **7a** .......... 3

**Figure S2:** $^{13}$C NMR spectrum of compound **7a** .......... 3

**Figure S3:** $^{1}$H NMR spectrum of compound **7b** .......... 4

**Figure S4:** $^{13}$C NMR spectrum of compound **7b** .......... 5

**Figure S5:** $^{1}$H NMR spectrum of compound **7c** .......... 5

**Figure S6:** $^{13}$C NMR spectrum of compound **7c** .......... 5

**Figure S7:** $^{1}$H NMR spectrum of compound **7d** .......... 6

**Figure S8:** $^{13}$ CNMR spectrum of compound **7d** .......... 6

**Figure S9:** $^{1}$H NMR spectrum of compound **7e** .......... 7

**Figure S10:** $^{13}$C NMR spectrum of compound **7e** .......... 7

**Figure S11:** $^{1}$H NMR spectrum of compound **7f** .......... 8

**Figure S12:** $^{13}$C NMR spectrum of compound **7f** .......... 8

**Figure S13**: $^{1}$H NMR spectrum of compound **7g** .......... 9

**Figure S14:** $^{13}$C NMR spectrum of compound **7g** .......... 9

**Figure S15:** $^{1}$H NMR spectrum of compound **7h** .......... 10

**Figure S16:** $^{1}$H NMR spectrum of compound **7i** ..... 10

**Figure S17:** $^{13}$ CNMR spectrum of compound **7i** ..... 11

**Figure S18:** $^{1}$H NMR spectrum of compound **7j** ..... 11

Figure S19: $^{13}$C NMR spectrum of compound 7j ..... 12

**Figure S20:** $^{1}$H NMR spectrum of compound **7k** ..... 12

**Figure S21:** $^{13}$C NMR spectrum of compound **7k** ..... 13

**Figure S22:** $^{1}$H NMR spectrum of compound **7l** ..... 13

**Figure S23**: $^{13}$C NMR spectrum of compound **7l** ..... 14

**Figure S24:** $^{1}$H NMR spectrum of compound **7m** ..... 14

**Figure S25**: $^{13}$C NMR spectrum of compound **7m** ..... 15

**Figure S26:** Evaluation of antiviral activity of the compounds against yellow fever, Herpes simplex and zika virus ..... 15

**Figure S27**: Representative numbering for compounds **7m** and **7k** ..... 16

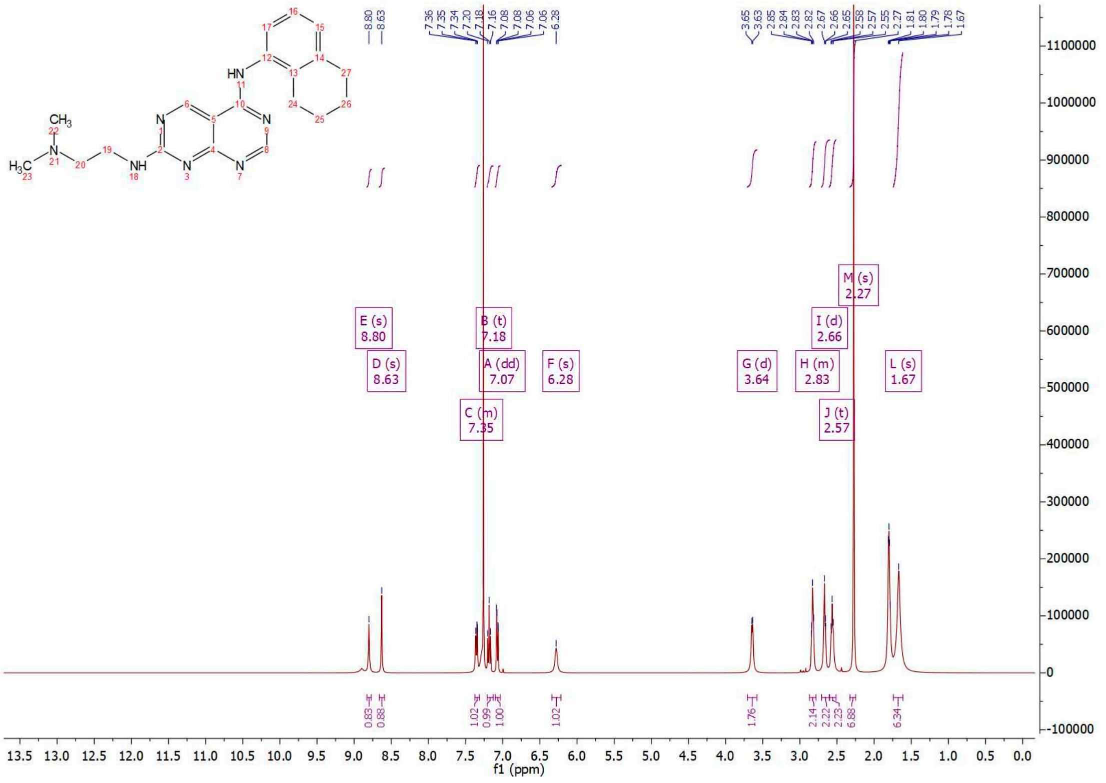

Figure S1: $^{1}$H NMR spectrum of compound **7a**

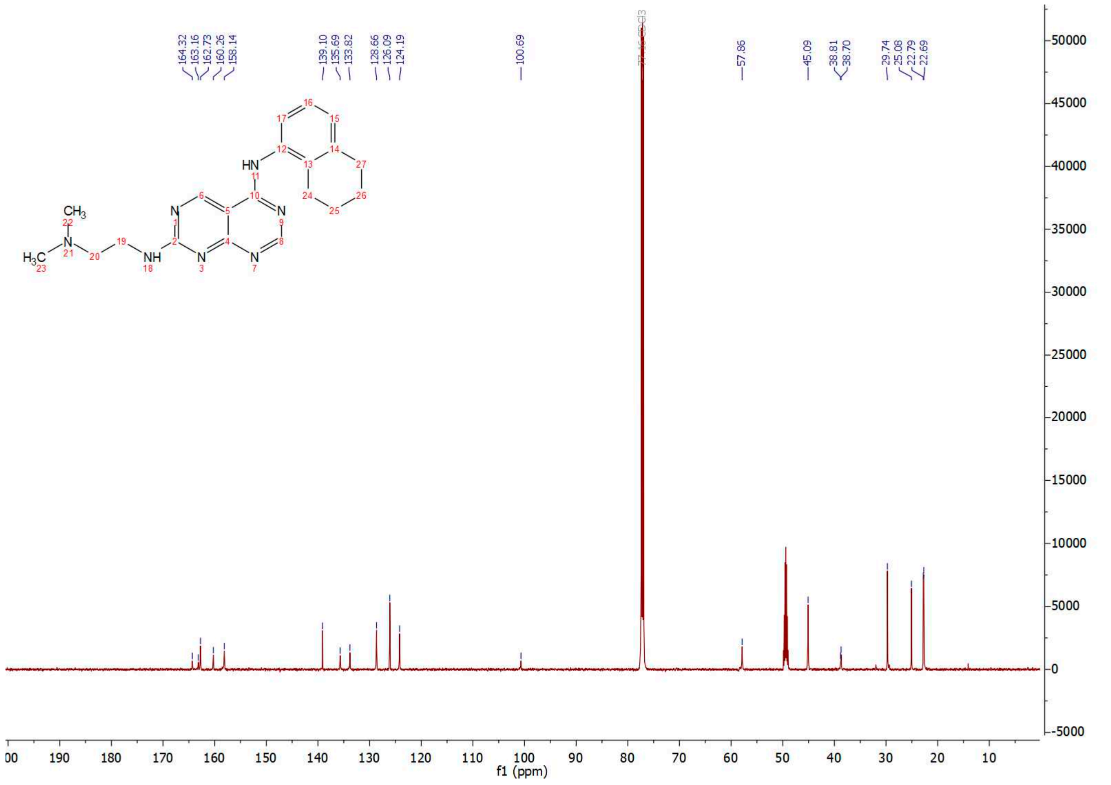

Figure S2: $^{13}$C NMR spectrum of compound **7a**

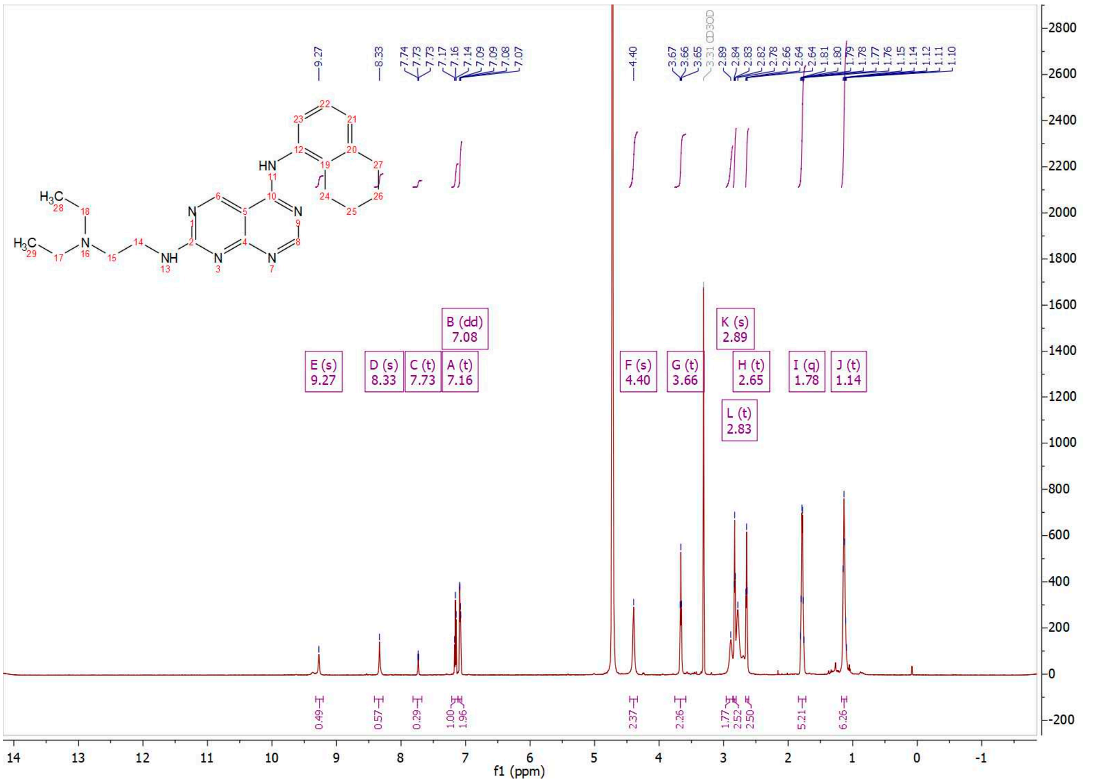


Figure S3: $^{1}$H NMR spectrum of compound **7b**

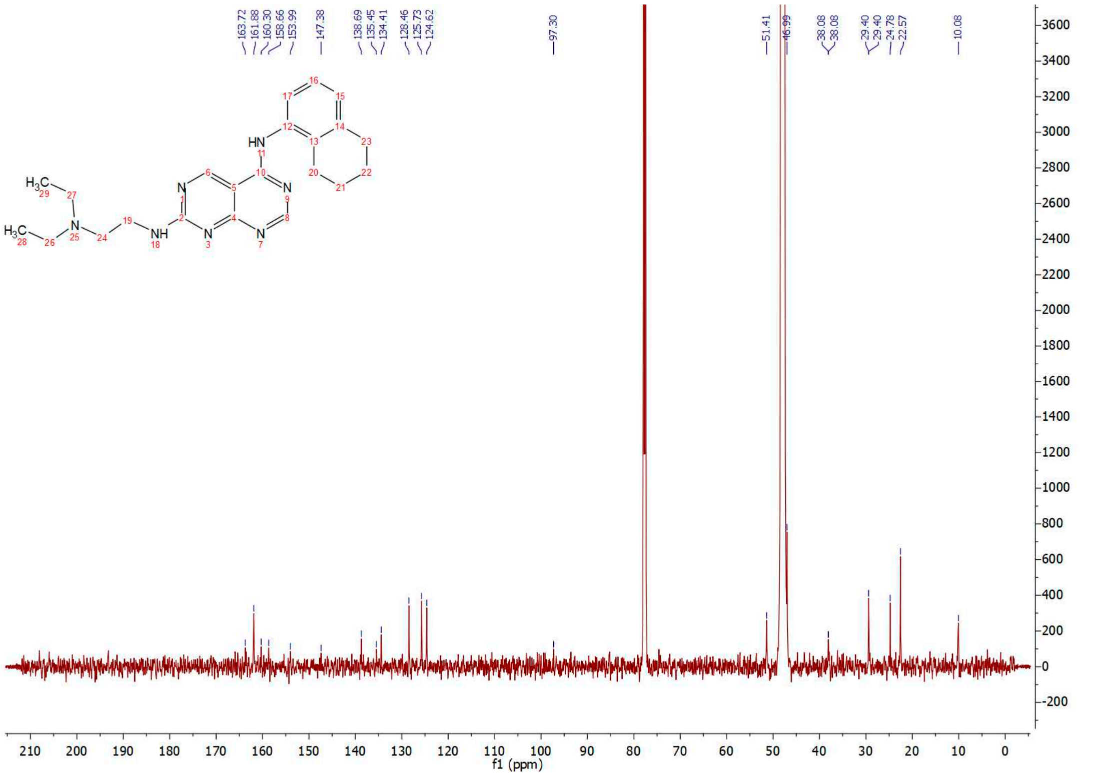


Figure S4: $^{13}$C NMR spectrum of compound **7b**

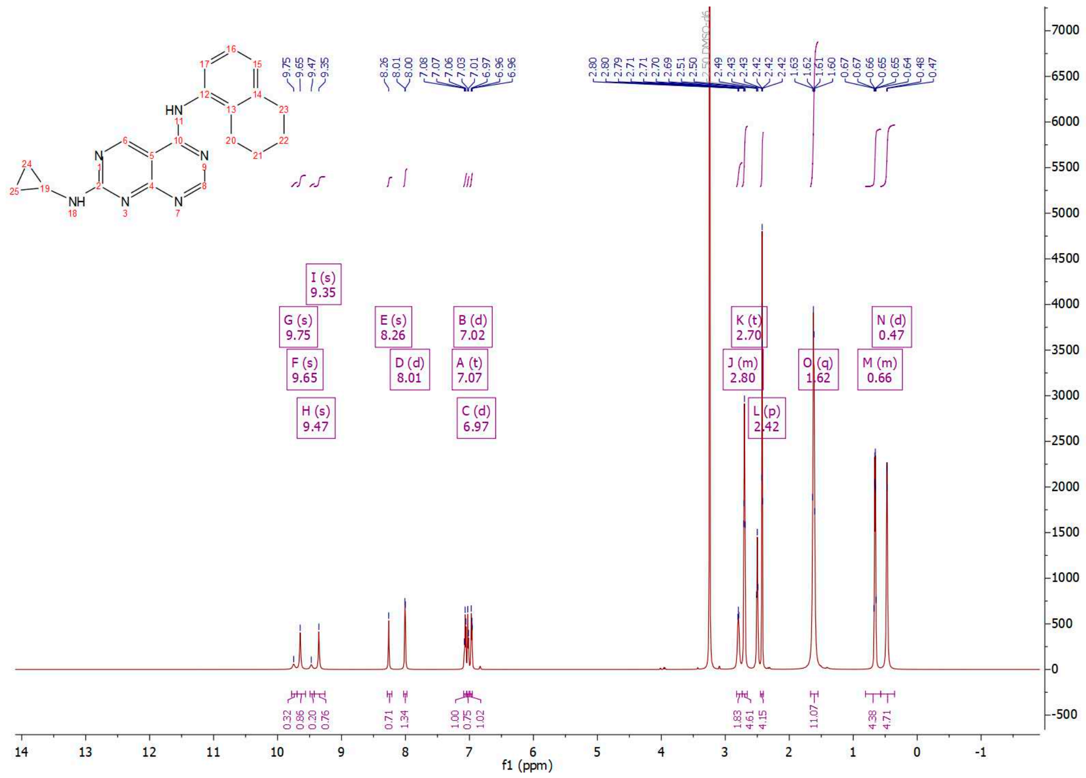

Figure S5: $^{1}$H NMR spectrum of compound **7c**

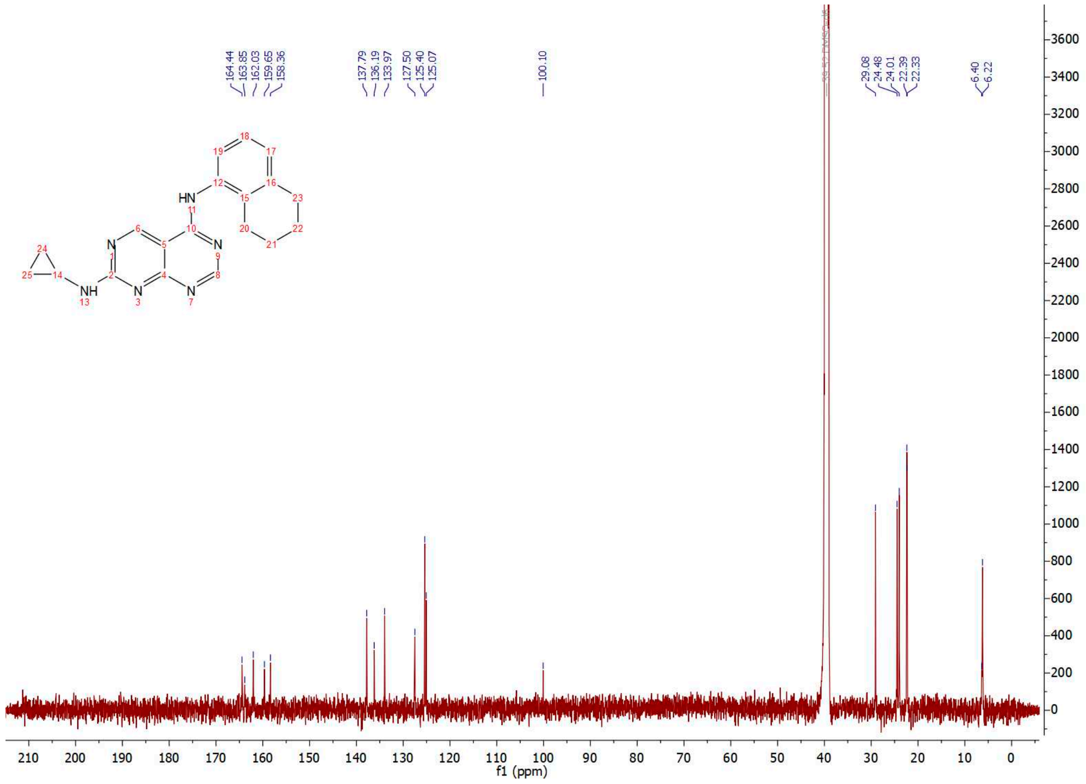

Figure S6: $^{13}$C NMR spectrum of compound **7c**

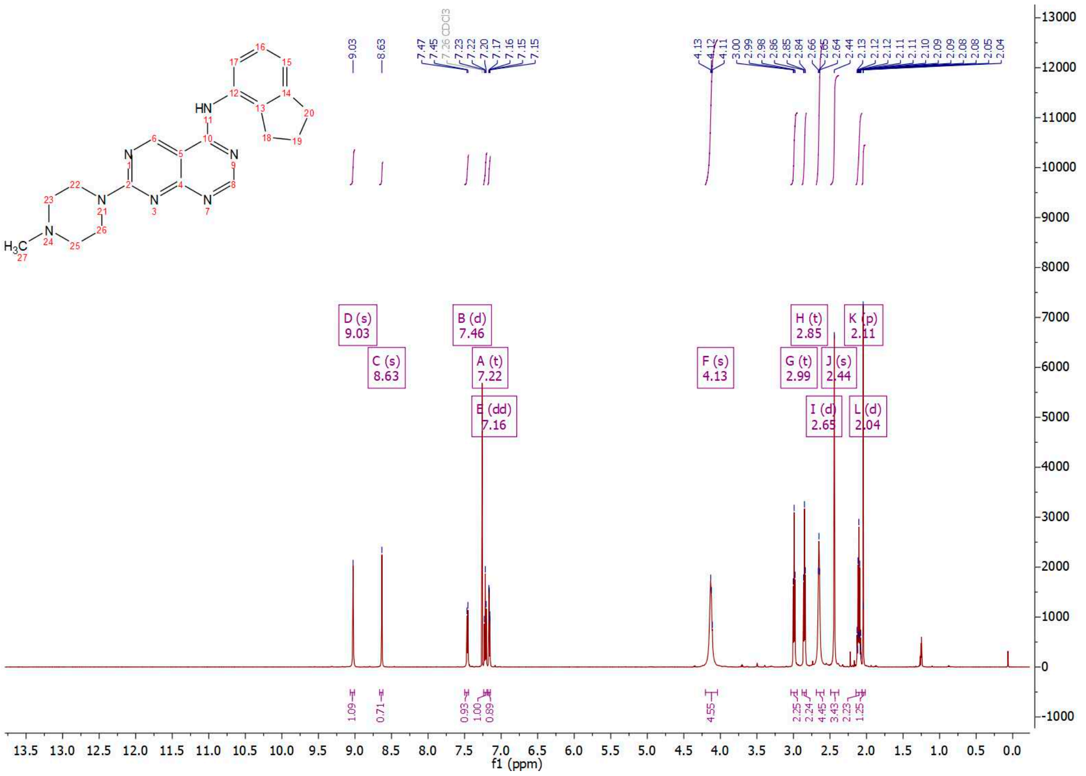

Figure S7: $^{1}$H NMR spectrum of compound **7d**

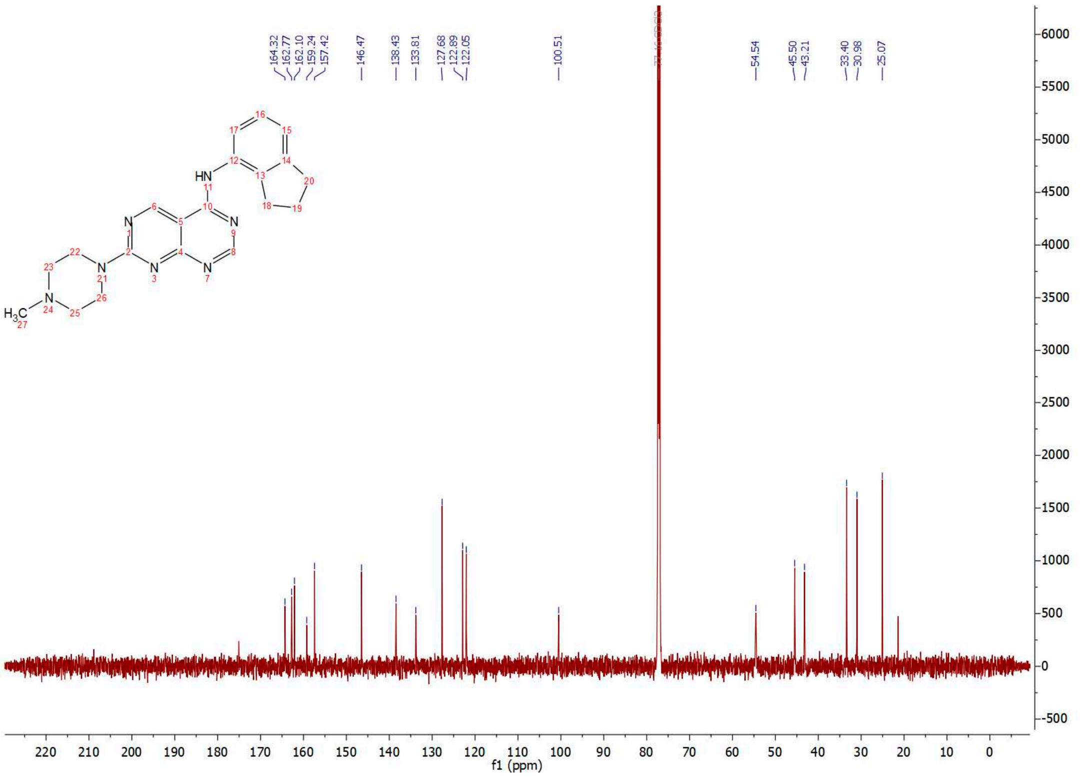

Figure S8: $^{13}$CNMR spectrum of compound **7d**

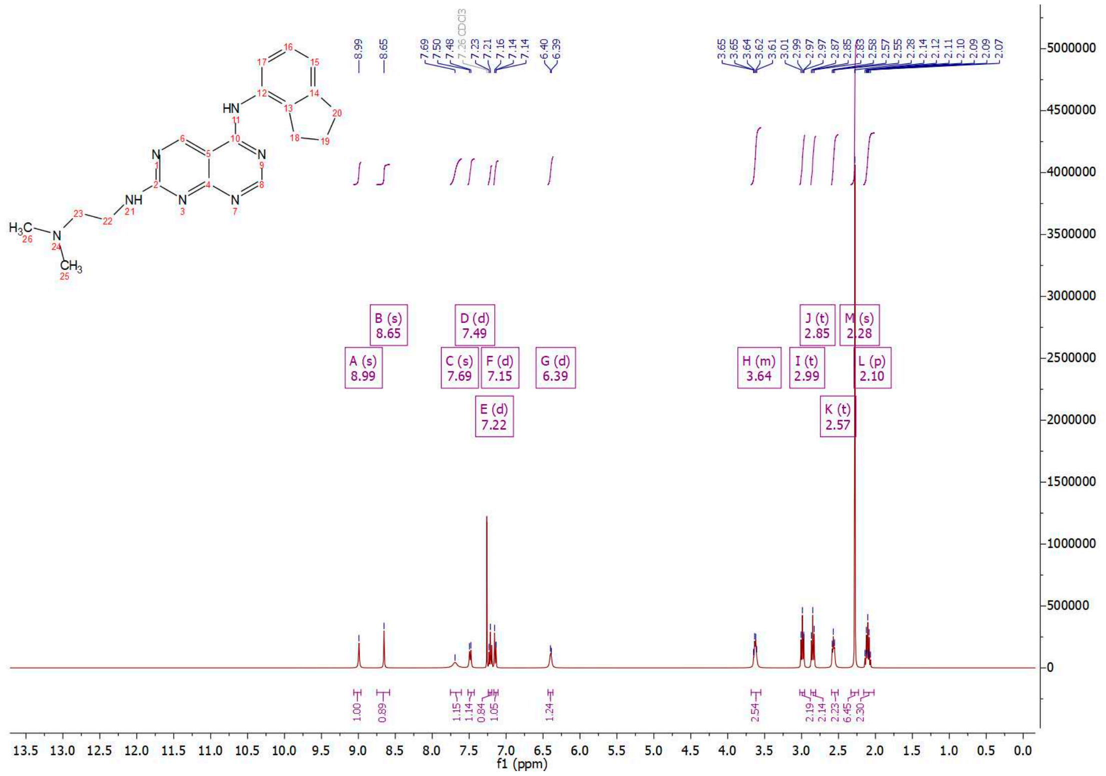

Figure S9: $^{1}$H NMR spectrum of compound **7e**

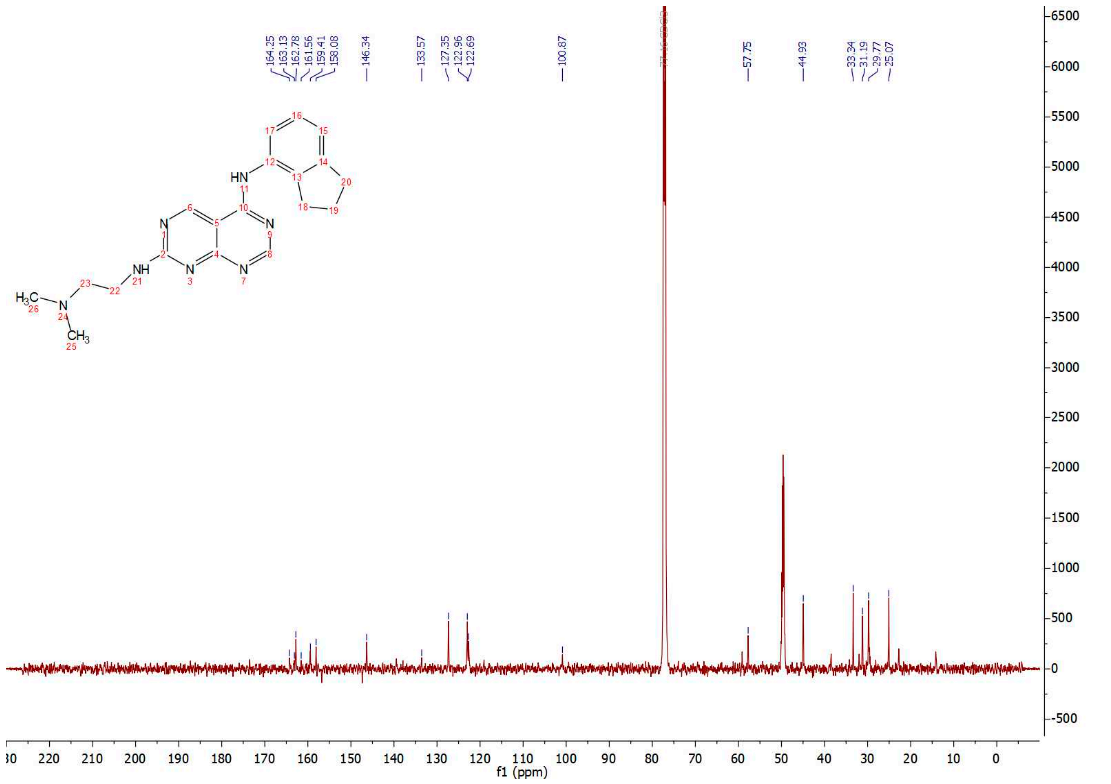

Figure S10: $^{13}$C NMR spectrum of compound **7e**

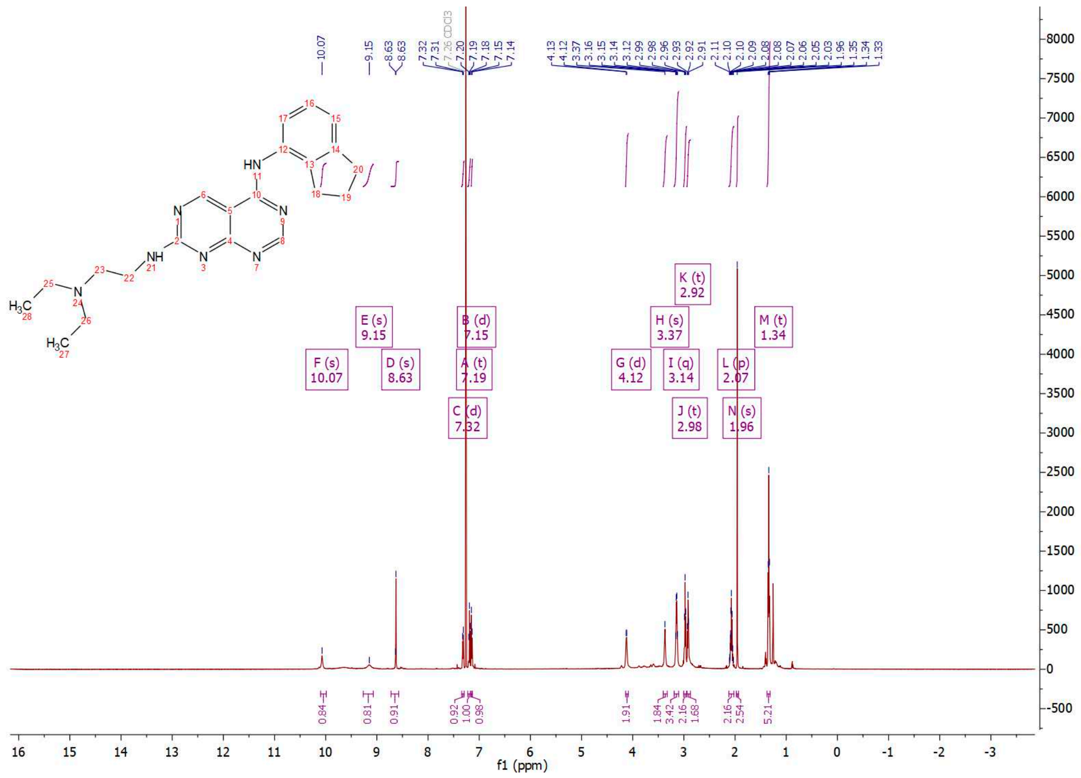

Figure S11: $^{1}H$ NMR spectrum of compound **7f**

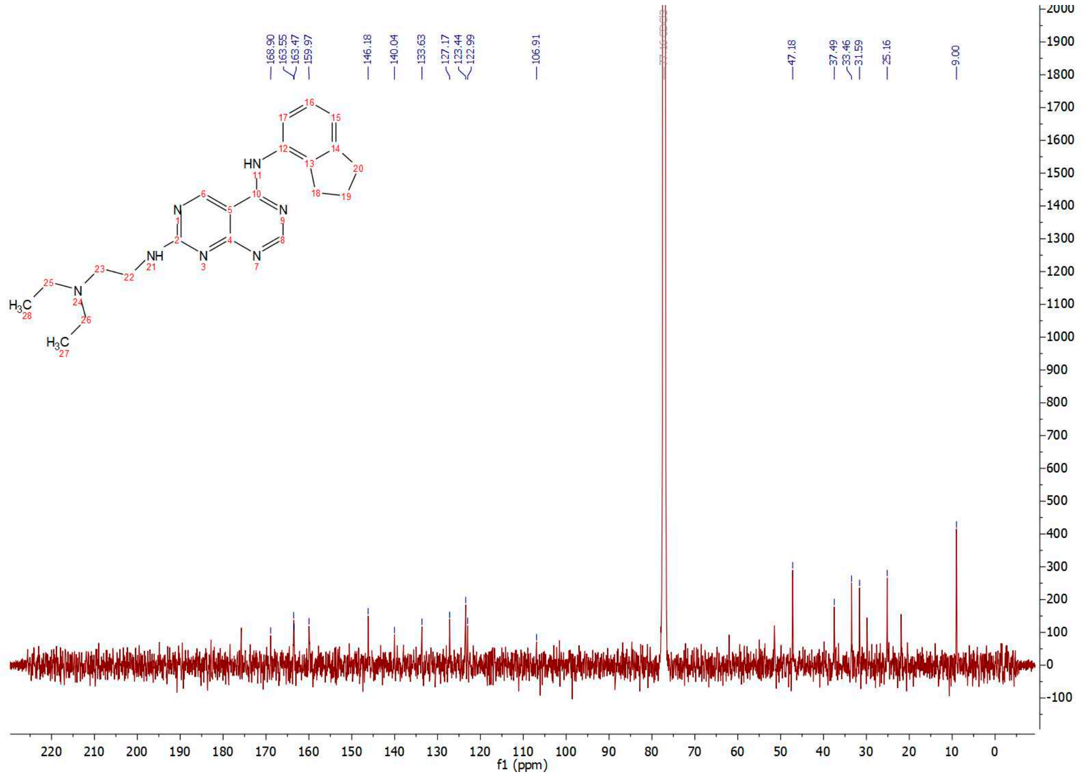

Figure S12: $^{13}C$ NMR spectrum of compound **7f**

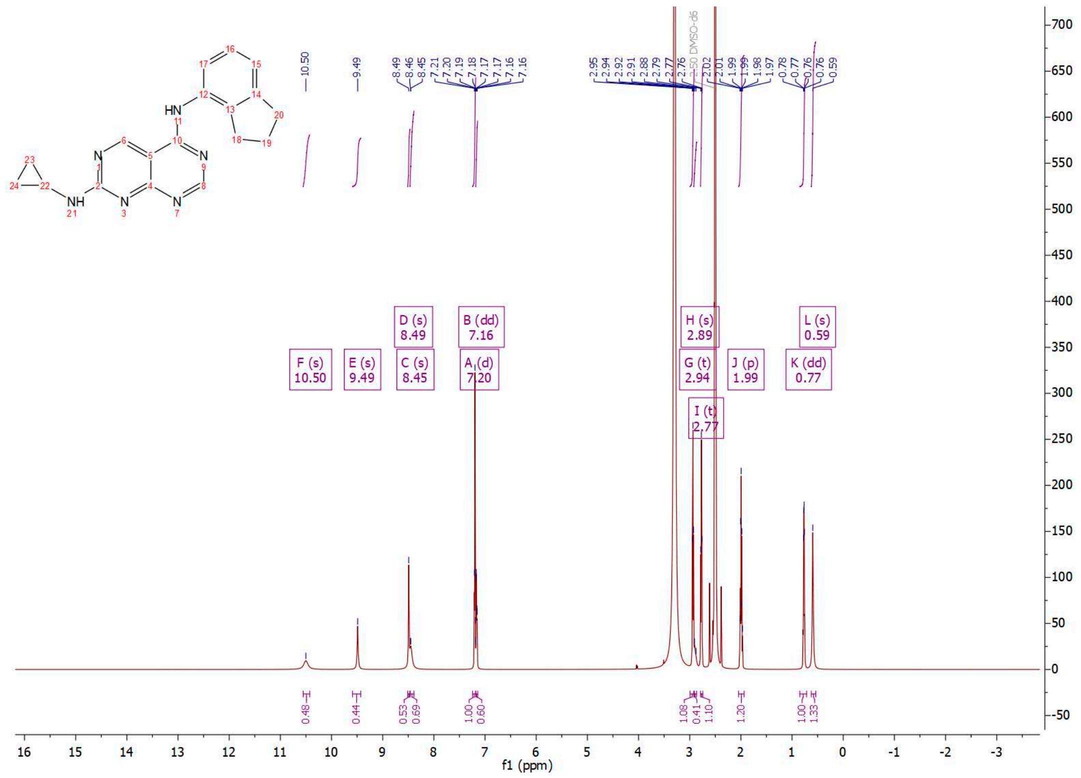

Figure S13: ¹H NMR spectrum of compound **7g**

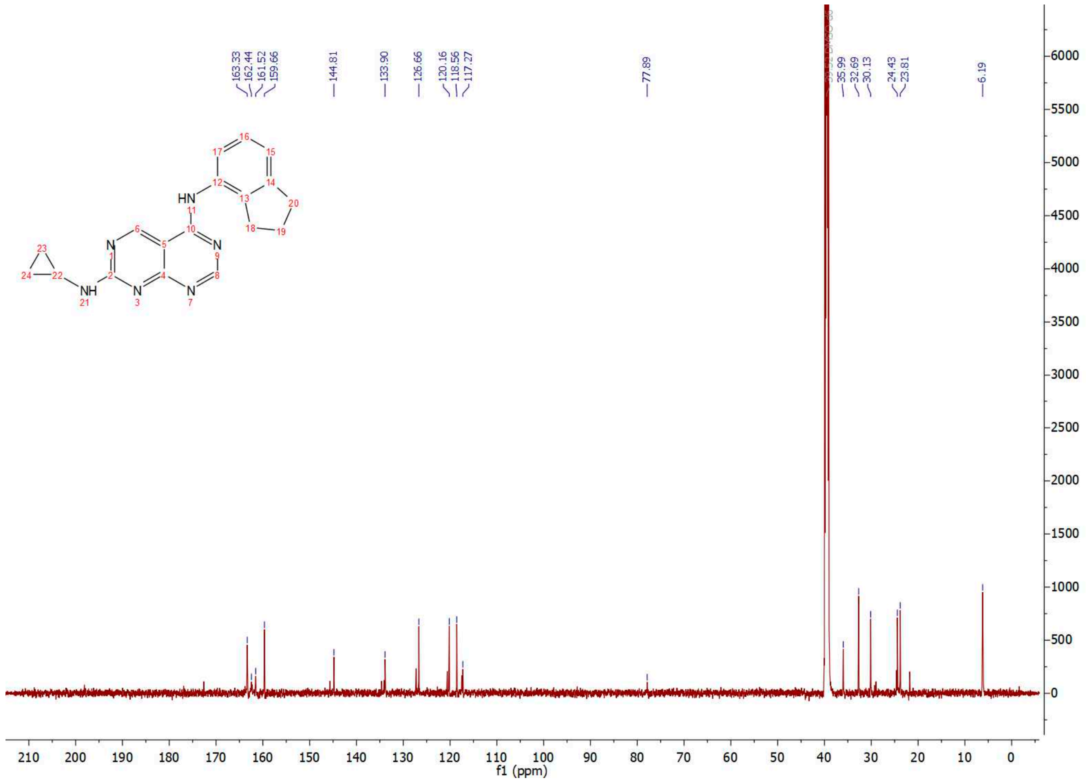

Figure S14: ¹³C NMR spectrum of compound **7g**

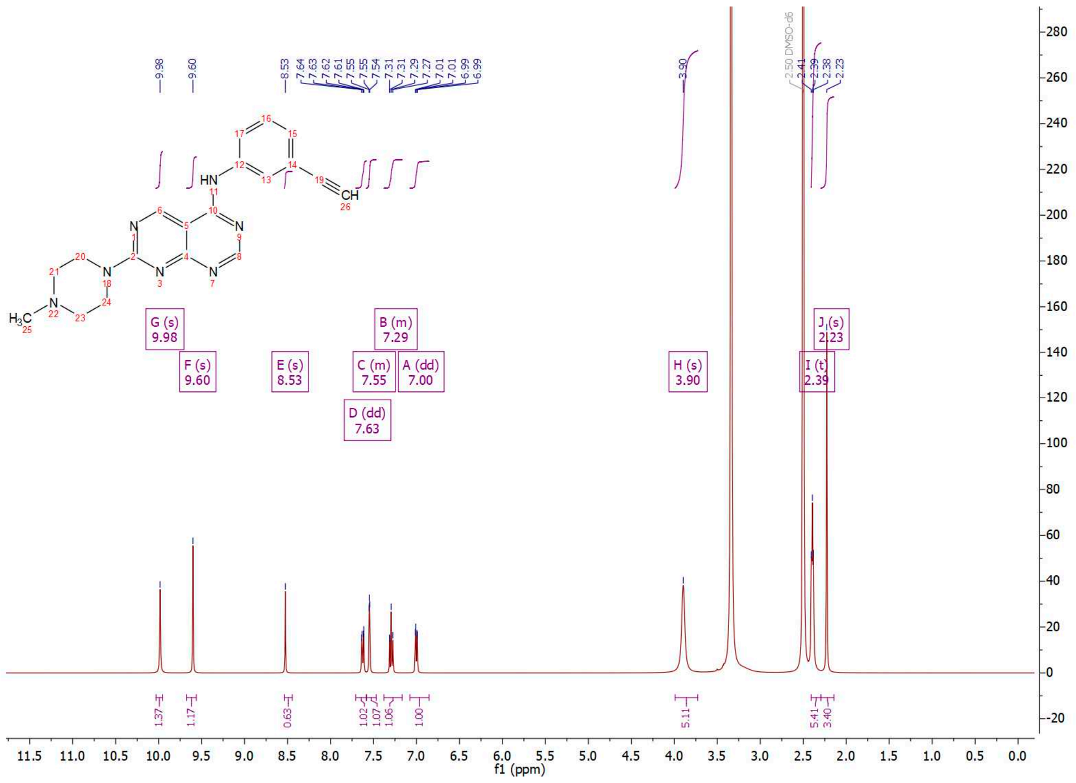

Figure S15: $^{1}$H NMR spectrum of compound **7h**

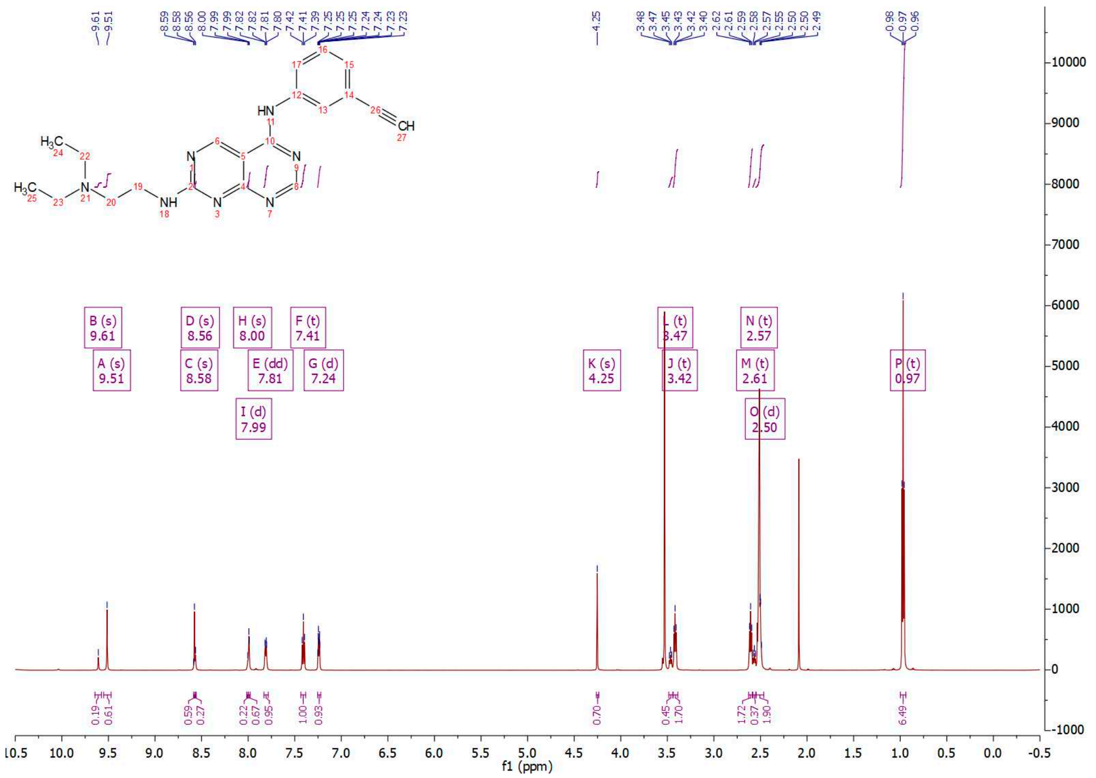

Figure S16: $^{1}$H NMR spectrum of compound **7i**

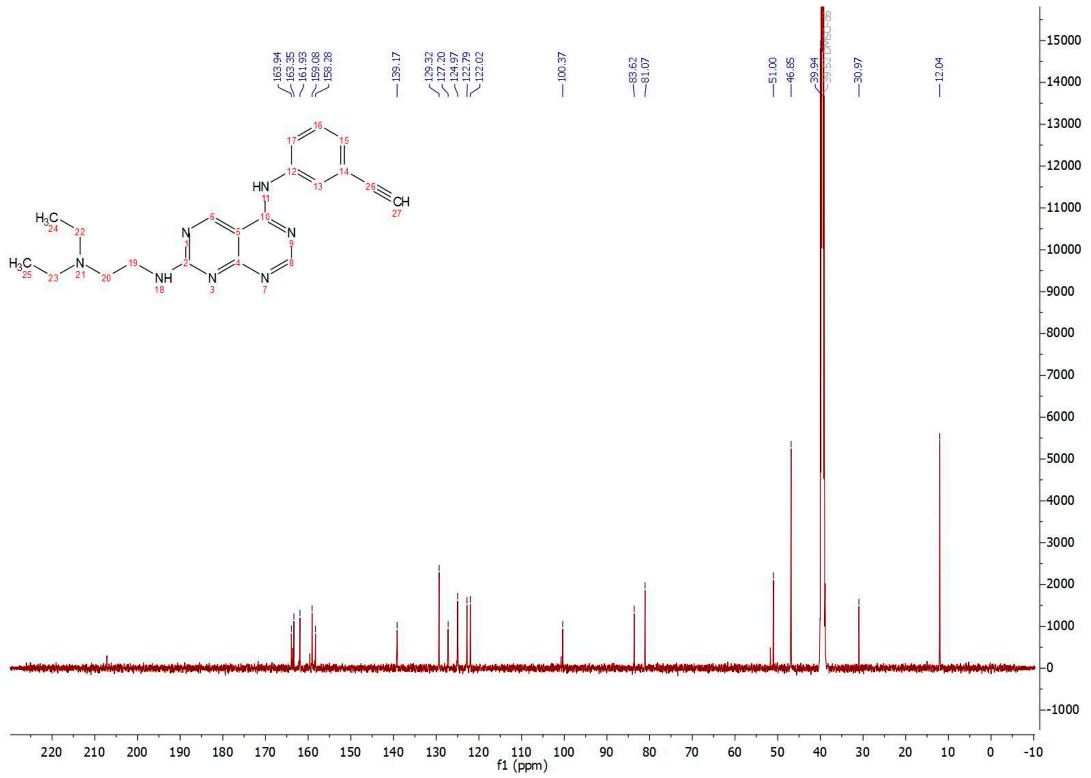

Figure S17: [13] CNMR spectrum of compound **7i**

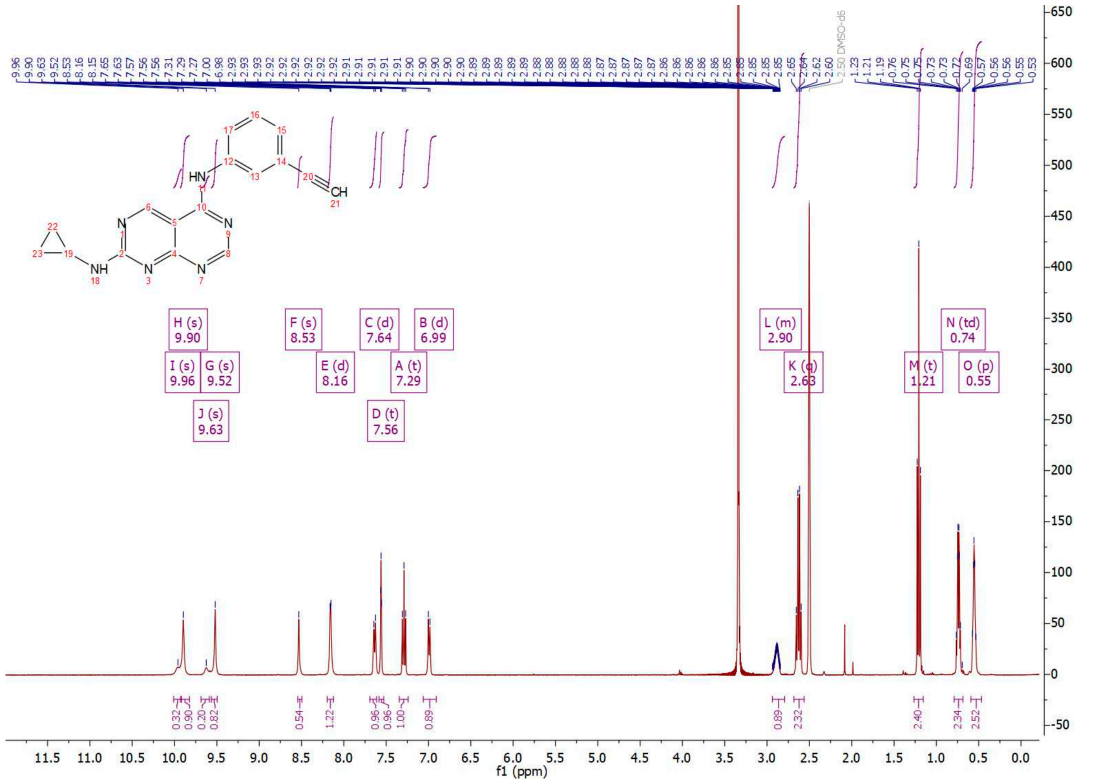

Figure S18: $^{1}$H NMR spectrum of compound **7j**

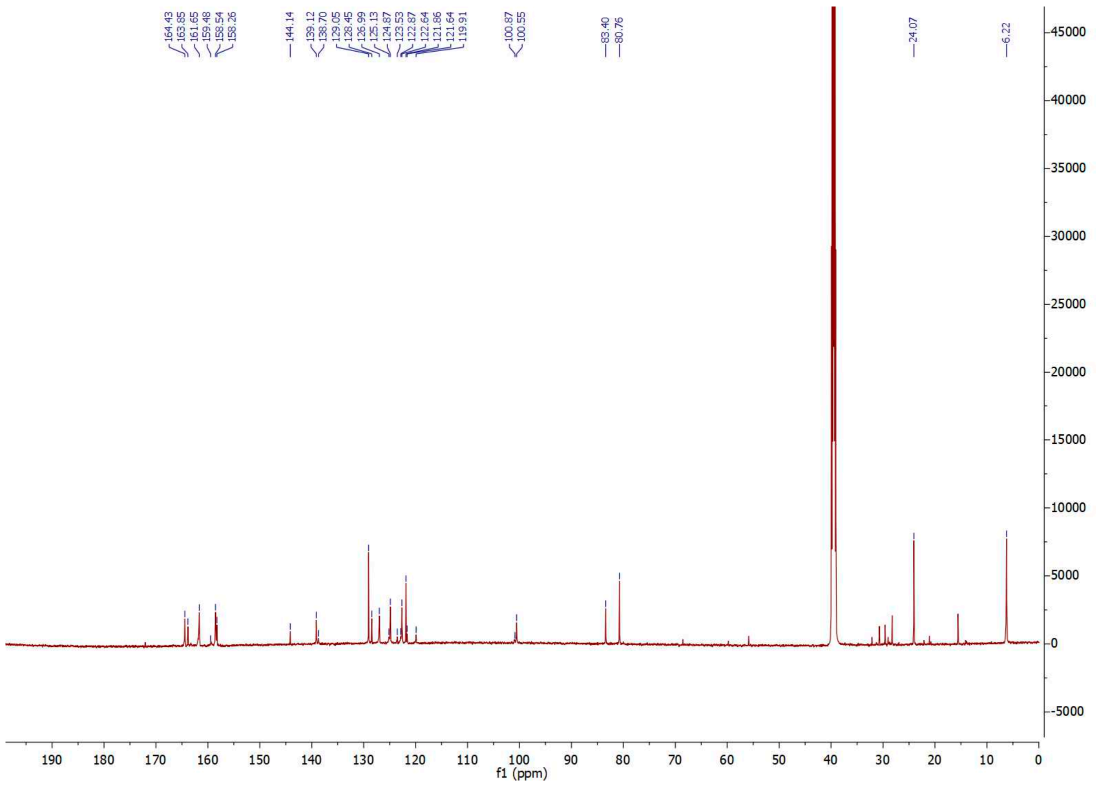

Figure S19: $^{13}$C NMR spectrum of compound 7j

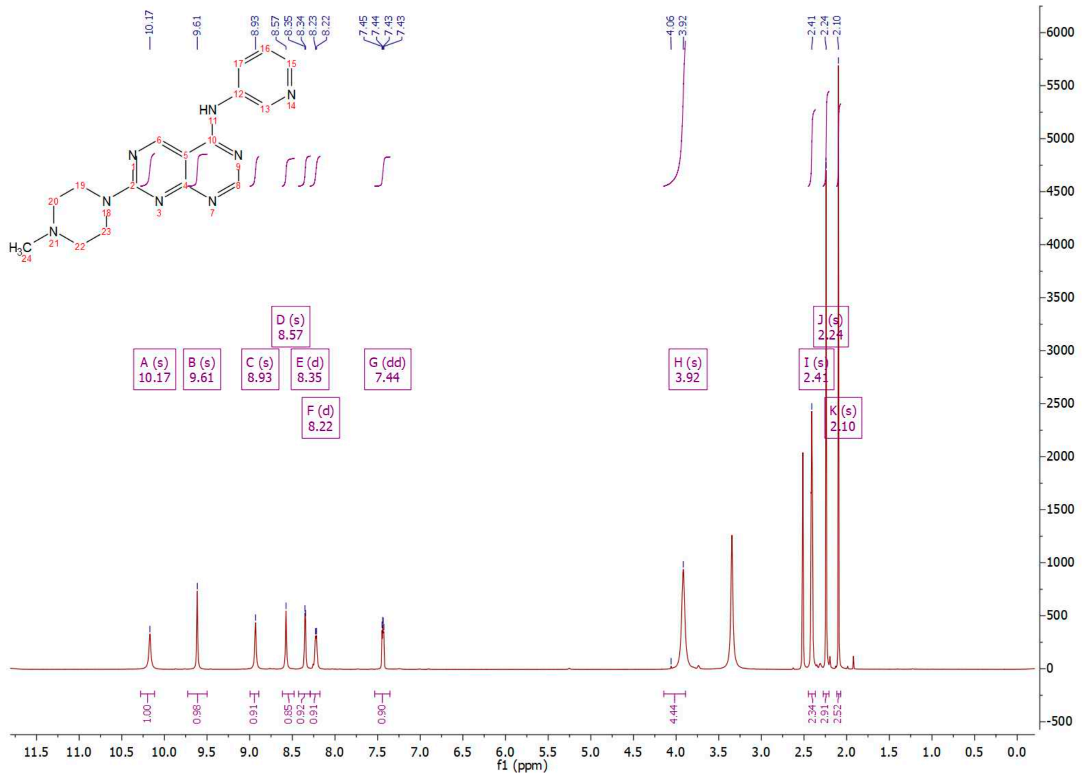

Figure S20: $^{1}$H NMR spectrum of compound **7k**

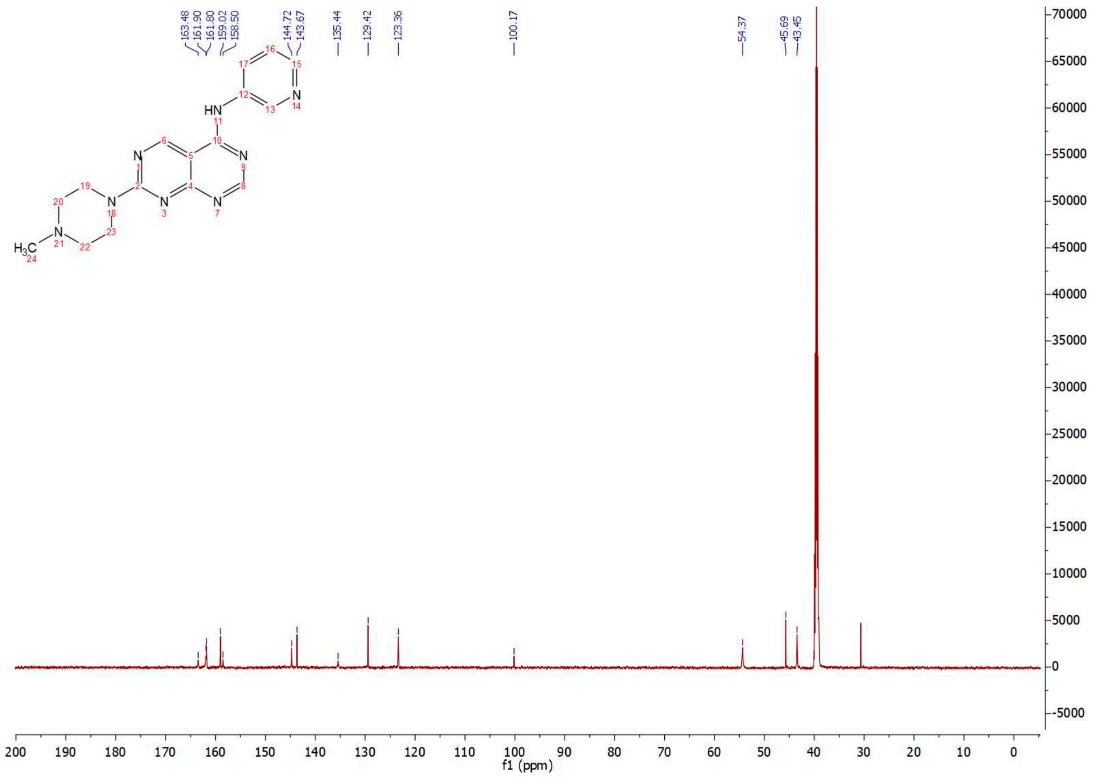


Figure S21: $^{13}C$ NMR spectrum of compound **7k**

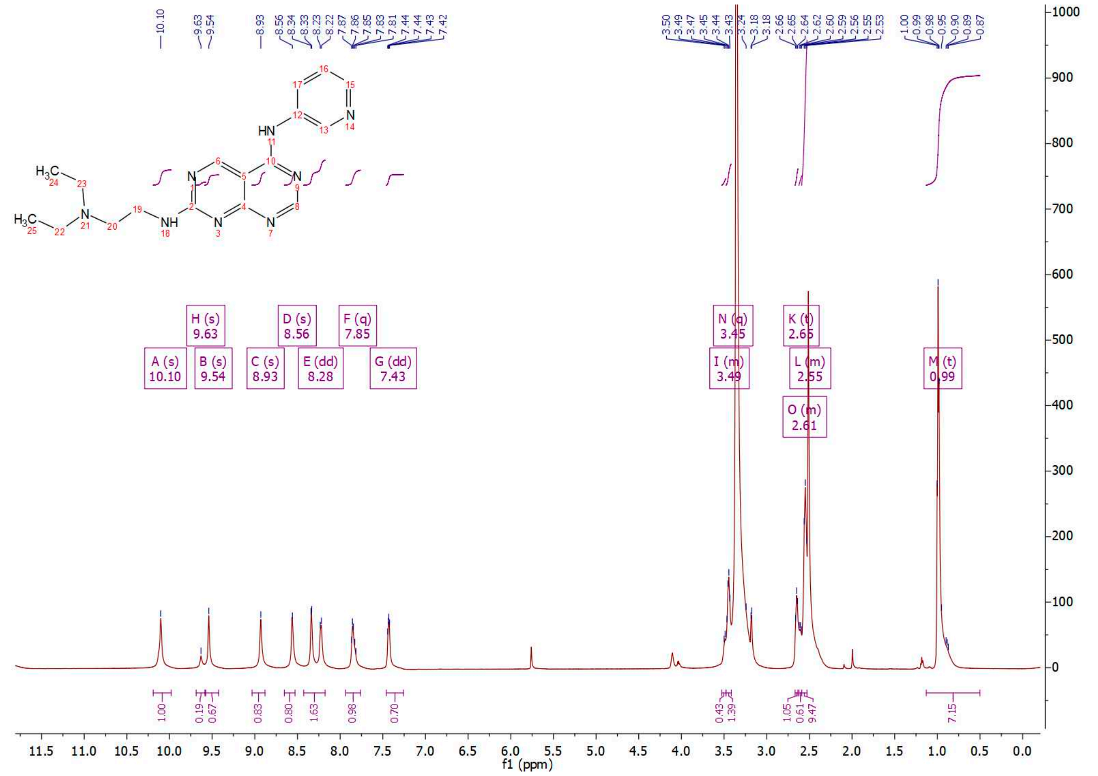


Figure S22: $^{1}H$ NMR spectrum of compound **7l**

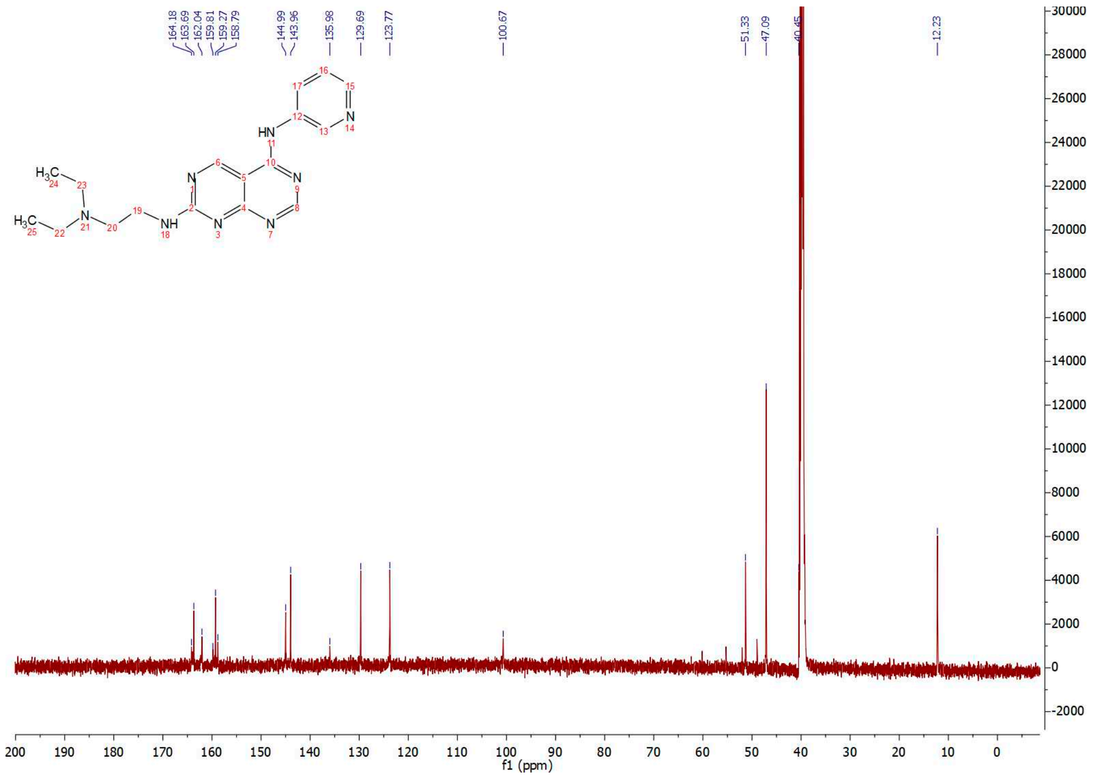

Figure S23: $^{13}C$ NMR spectrum of compound **7l**

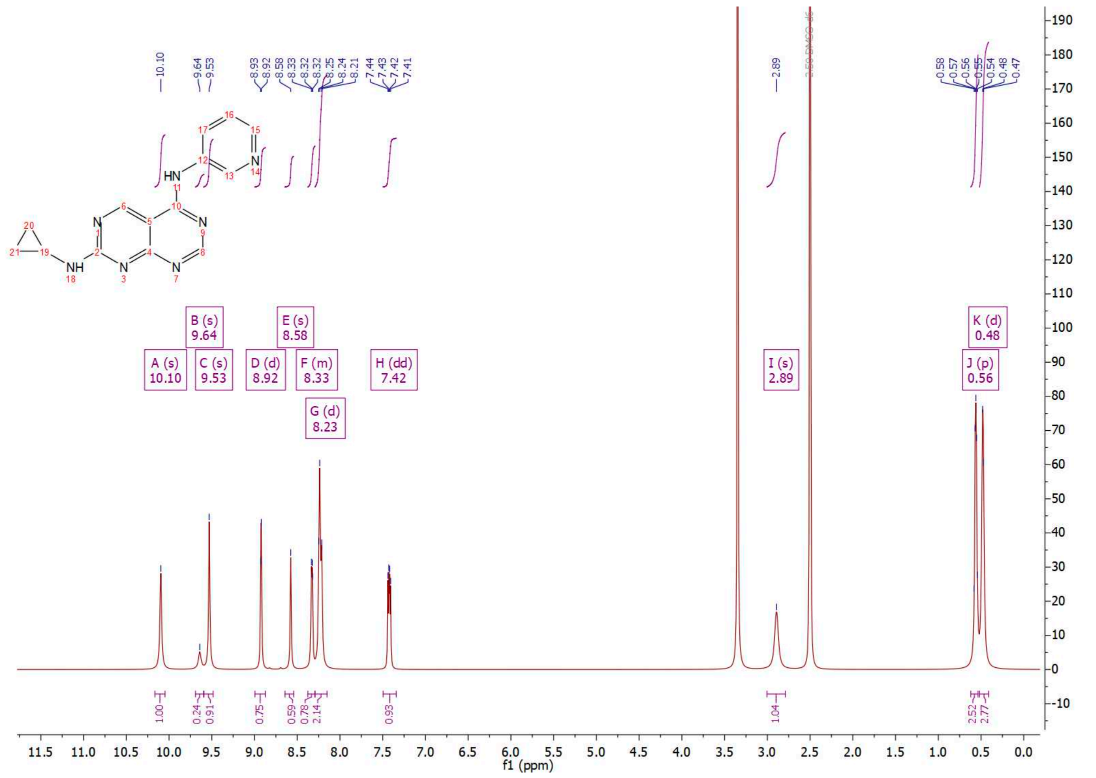

Figure S24: $^{1}H$ NMR spectrum of compound **7m**

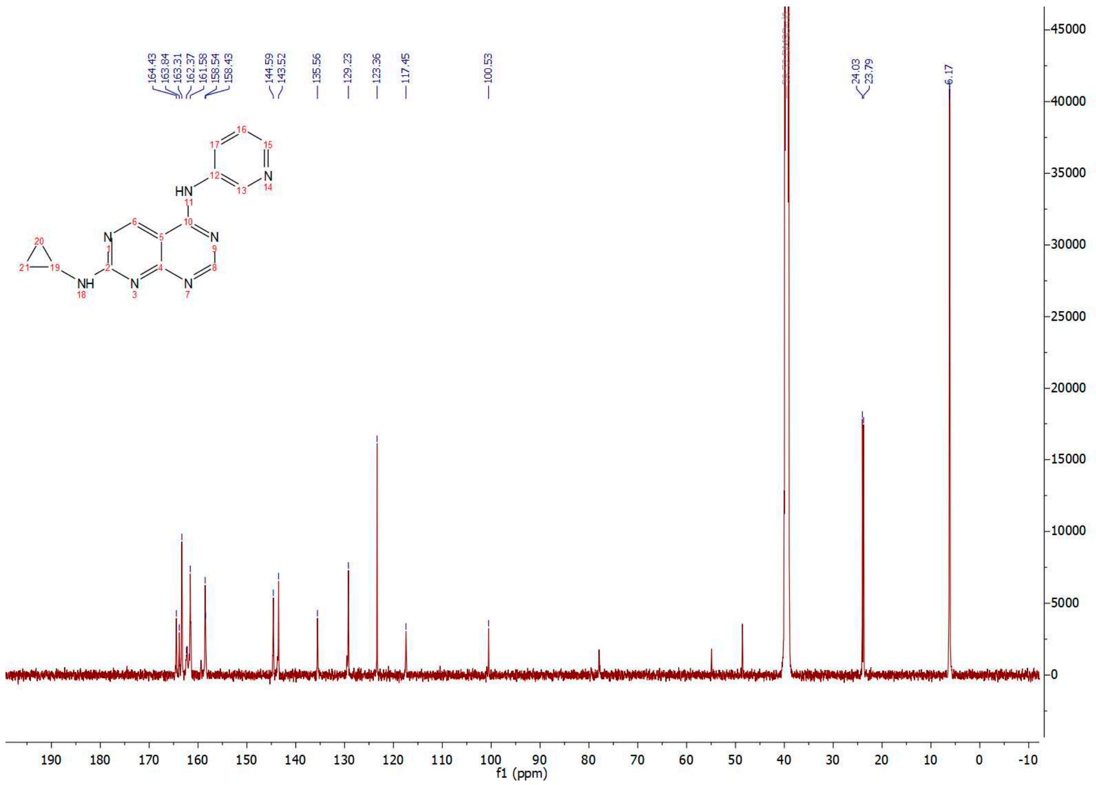


Figure S25: $^{13}$C NMR spectrum of compound **7m**

| Compd. | HSV-1 KOS HEL 299 | YFV 17D VeroE6 | ZikV mr766 VeroE6 | Cytotoxicity ($CC_{50}$) HEL 299 | Hep3B | MDCK | Hep2 | VeroE6 |
|---|---|---|---|---|---|---|---|---|
| **7a** | >50 | >50 | >50 | >50 | >50 | >50 | >50 | >50 |
| **7b** | >50 | >50 | >50 | >50 | >50 | >50 | >50 | >50 |
| **7c** | >50 | >50 | >50 | >50 | >50 | >50 | >50 | >50 |
| **7d** | >50 | >50 | >50 | >50 | >50 | >50 | >50 | >50 |
| **7e** | >50 | >50 | >50 | >50 | >50 | >50 | >50 | >50 |
| **7f** | >50 | >50 | >50 | >50 | >50 | >50 | >50 | >50 |
| **7g** | >50 | >50 | >50 | >50 | >50 | >50 | >50 | >50 |
| **7h** | >50 | >50 | >50 | >50 | >50 | >50 | 29,6 ± 1,6 | >50 |
| **7i** | >50 | >50 | >50 | >50 | 25,7 ± 0,6 | >50 | >50 | >50 |
| **7j** | >50 | >50 | >50 | >50 | >50 | >50 | >50 | >50 |
| **7k** | >50 | >50 | >50 | >50 | >50 | >50 | >50 | >50 |
| **7l** | >50 | >50 | >50 | >50 | >50 | >50 | >50 | >50 |
| **7m** | >50 | >50 | >50 | >50 | >50 | >50 | >50 | >50 |
| Erlotinib | >100 | >100 | >100 | >100 | >100 | >100 | >100 | >100 |
| WZ4002 | >100 | >100 | >100 | >100 | >100 | >100 | >100 | >100 |
| OSI-420 | >100 | >100 | >100 | >100 | >100 | >100 | 41,0 ± 13,4 | >100 |
| Icotinib | >100 | >100 | >100 | >100 | >100 | >100 | >100 | >100 |
| CNX-2006 | >100 | >100 | >100 | 11,1 ± 1,0 | 4,3 ± 1,8 | 1,7 ± 0,4 | 11,7 ± 0,8 | 9,2 ± 1,1 |
| CL-387785 | 1,0 ± 0,2 | >100 | >100 | >100 | 10,9 ± 0,6 | 7,1 ± 0,4 | 10,4 ± 1,9 | >100 |
| Remdesivir | - | - | - | >10 | 8,1 ± 2,5 | - | 9,0 ± 1,3 | - |
| Ribavirin | - | 24,5 ± 3,8 | 103,6 ± 12,3 | >250 | 165,1 ± 20,2 | >250 | 88,8 ± 23,5 | >250 |
| Zanamivir | - | - | - | - | - | >100 | - | - |
| Rimantadine | - | - | - | - | - | >100 | - | - |

Data are presented as mean ± standard deviation (SD)

Figure S26: Evaluation of antiviral activity of the compounds against yellow fever, Herpes simplex and zika virus

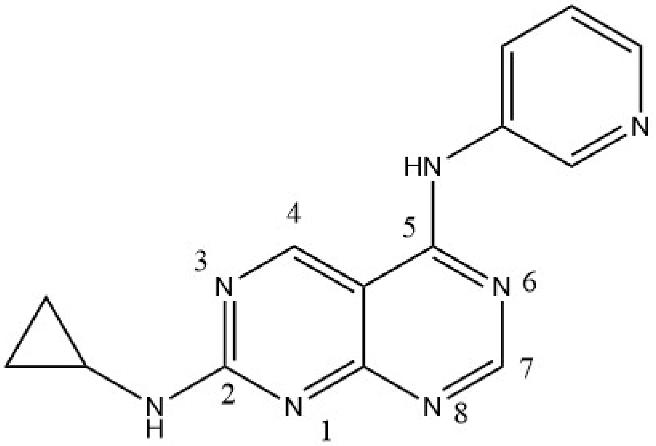


N2-cyclopropyl-N5-(pyridin-3-yl)pyrimido[4,5-d]pyrimidine-2,5-diamine **7m**

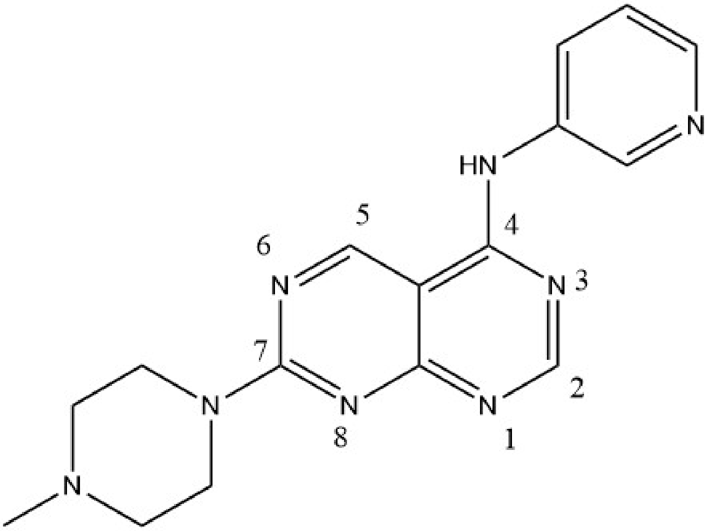


7-(4-methylpiperazin-1-yl)-N-(pyridin-3-yl)pyrimido[4,5-d]pyrimidin-4-amine **7k**

Figure S27: Representative numbering for compounds 7m and 7k
